# Unveiling the powerhouse: ASCL1-driven small cell lung cancer is characterized by higher numbers of mitochondria and enhanced oxidative phosphorylation

**DOI:** 10.1186/s40170-025-00382-6

**Published:** 2025-03-31

**Authors:** Anna Solta, Büsra Ernhofer, Kristiina Boettiger, Christian Lang, Zsolt Megyesfalvi, Theresa Mendrina, Dominik Kirchhofer, Gerald Timelthaler, Beata Szeitz, Melinda Rezeli, Clemens Aigner, Arvand Haschemi, Lukas W. Unger, Balazs Dome, Karin Schelch

**Affiliations:** 1 Department of Thoracic Surgery, Comprehensive Cancer Center, Medical University of Vienna, Waehringer Guertel 18-20, Vienna, A-1090 Austria; 2 Division of Pulmonology, Department of Medicine II, Medical University of Vienna, Vienna, Austria; 3 Department of Thoracic Surgery, Semmelweis University and National Institute of Oncology, Budapest, Hungary; 4 National Koranyi Institute of Pulmonology, Budapest, Hungary; 5 Center for Cancer Research, Medical University of Vienna, Vienna, Austria; 6 Institute of Inorganic Chemistry, Faculty of Chemistry, University of Vienna, Vienna, Austria; 7 Department of Biomedical Engineering, Lund University, Lund, Sweden; 8 Department of Laboratory Medicine, Medical University of Vienna, Vienna, Austria; 9 Deptartment of Colorectal Surgery, Oxford University Hospitals, Oxford, UK; 10 Division of Visceral Surgery, Department of General Surgery, Medical University of Vienna, Vienna, Austria; 11 Department of Translational Medicine, Lund University, Lund, Sweden

**Keywords:** Small cell lung cancer, Metabolism, Oxidative phosphorylation, Molecular subtypes

## Abstract

**Background:**

Small cell lung cancer (SCLC) is an aggressive malignancy with distinct molecular subtypes defined by transcription factors and inflammatory characteristics. This follow-up study aimed to validate the unique metabolic phenotype in achaete-scute homologue 1 (ASCL1)-driven SCLC cell lines and human tumor tissue.

**Methods:**

Metabolic alterations were analyzed using proteomic data. Structural and functional differences of mitochondria were investigated using qPCR, flow cytometry, confocal imaging, and transmission electron microscopy and seahorse assays. Several metabolic inhibitors were tested using MTT-based and clonogenic assays. Single-cell enzyme activity assays were conducted on cell lines and tumor tissue samples of SCLC patients.

**Results:**

We found increased mitochondrial numbers correlating with higher oxidative phosphorylation activity in ASCL1-dominant cells compared to other SCLC subtypes. Metabolic inhibitors targeting mitochondrial respiratory complex-I or carnitine palmitoyltransferase 1 revealed higher responsiveness in SCLC-A. Conversely, we demonstrated that non-ASCL1-driven SCLCs with lower oxidative signatures show dependence on glutaminolysis as evidenced by the enhanced susceptibility to glutaminase inhibition. Accordingly, we detected increased glutamate-dehydrogenase activity in non-ASCL1-dominant cell lines as well as in human SCLC tissue samples.

**Conclusions:**

Distinct SCLC subtypes exhibit unique metabolic vulnerabilities, suggesting potential for subtype-specific therapies targeting the respiratory chain, fatty acid transport, or glutaminolysis.

**Graphical abstract:**

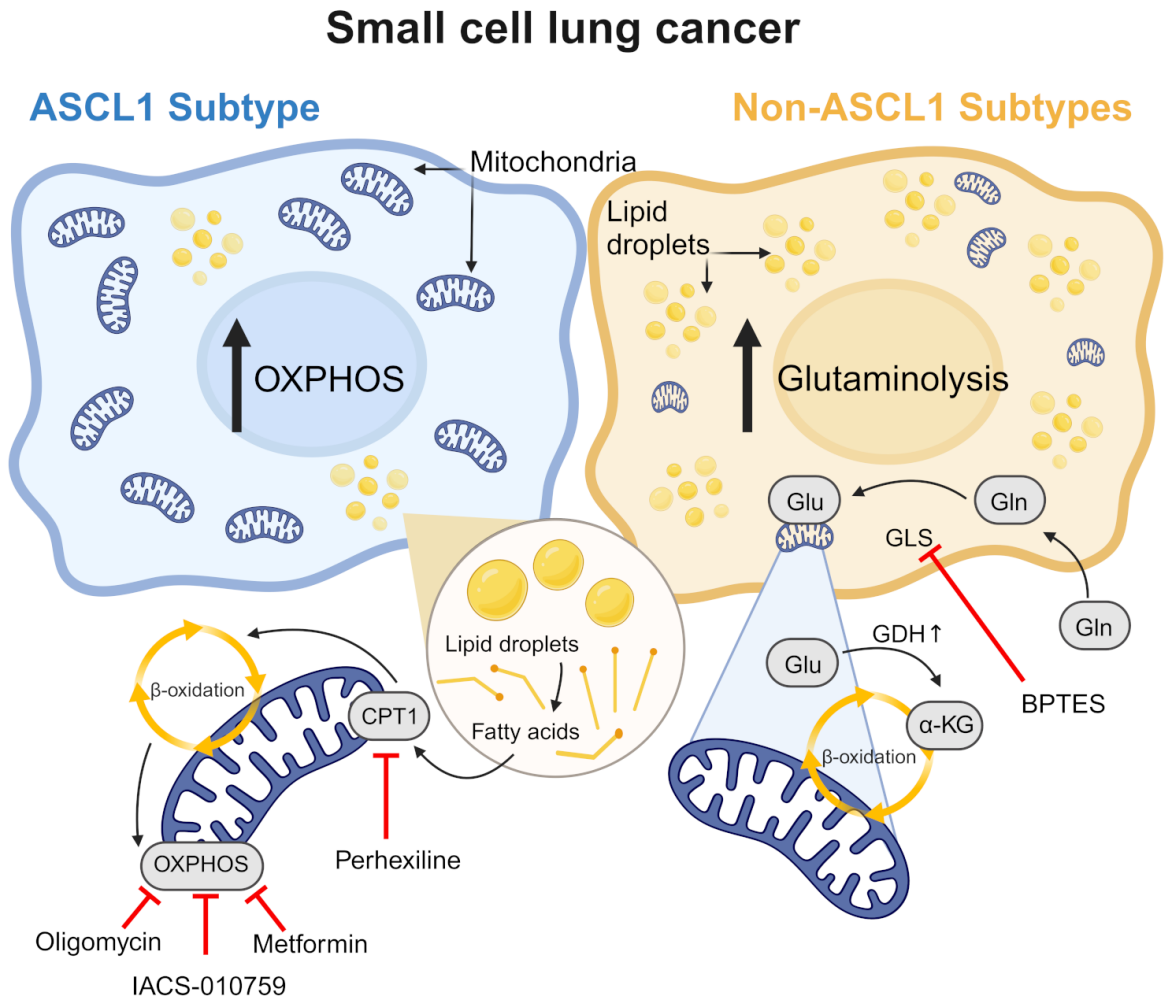

**Supplementary Information:**

The online version contains supplementary material available at 10.1186/s40170-025-00382-6.

## Introduction

Lung cancer remains the leading cause of cancer-related deaths worldwide. Approximately 13–15% of all lung cancer cases are classified as small cell lung cancer (SCLC) [1]. SCLC is characterized by an exceptionally poor prognosis, partly due to a high proliferation rate and early metastatic dissemination [2]. For decades, the standard of care therapeutic regimen for SCLC patients comprised a combination of platinum-based chemotherapy and etoposide [3]. The immune checkpoint inhibitors (ICIs) atezolizumab and durvalumab recently gained approval for the treatment of SCLC [4, 5]. From the molecular perspective, almost all SCLCs feature simultaneous inactivation of the genes *TP53* and *RB1*; however, being loss-of-function mutations, these inactivated tumor suppressors cannot be specifically targeted to date [6, 7].

Although several classification schemes have been proposed during the past few years in an attempt to define key expression profiles and corresponding vulnerabilities, current clinical treatment protocols are exclusively based on disease stage irrespective of the underlying molecular profile [8]. One recent preclinical classification of SCLC is based on the transcription regulators achaete-scute homologue 1 (ASCL1), neurogenic differentiation factor 1 (NEUROD1), POU class 2 homeobox 3 (POU2F3) and yes-associated protein 1 (YAP1) [8]. However, YAP1 was recently questioned by multiple authors regarding its independent role to define a SCLC subtype as validation studies could not identify YAP1 expressions in clinical samples [9, 10]. Concomitant ASCL1/ NEUROD1 (SCLC-AN) expressing tumors [10] as well as inflamed SCLCs have been recently described [1, 11, 12]. Importantly, high ASCL1 expression levels have been demonstrated to be independent negative prognosticators in surgically treated patients [9, 13].

Malignant transformation requires high amounts of energy. Therefore, metabolic reprogramming is one of the hallmarks of cancer. Various types of cancer, including lung or breast cancers, have been associated with the well-known Warburg effect [14]. This non-oxidative process is a very inefficient way of ATP production compared to aerobic oxidative phosphorylation [15]. Additional evidence indicates that metabolic reprogramming in cancer cells is fundamental to meet their high demand in building molecules with high-energy bonds such as ATP [16]. Overall, more recently published metabolic research highlights that cancer metabolism should be seen as a continuous process in which the reverse Warburg effect can equally take place, depending on the local milieu [17].

Recently, our group published distinct proteomic profiles of the SCLC molecular subtypes in cell lines characterized by the expression of ASCL1, NEUROD1, POU2F3, and YAP1 (SCLC-A/N/P/Y, respectively), suggesting oxidative phosphorylation as a unique signature for SCLC-A [18]. In the current study, we aimed to elucidate this unique metabolic phenotype of SCLC-A, shifting to oxidative phosphorylation and mitochondria-related processes including cellular response to fatty acids and other metabolites. To that aim, we performed structural and functional analysis of mitochondria in SCLC cell lines and human tumor tissue specimens. Here, we report differences in mitochondrial number and shape in addition to increased aerobic respiration between SCLC subtypes. We also demonstrate that SCLC cells associated with high ASCL1 expression are more susceptible to the blockage of mitochondrial respiratory complex I (NADH dehydrogenase) and, moreover, that non-ASCL1-driven cells are more susceptible to glutaminolysis inhibition, which may allow future subtype-specific personalized therapy.

## Materials and methods

### Cell culture and tissue specimens

All human SCLC cell lines used in this study were either obtained from collaborators or purchased from the American Type Culture Collection (ATCC; authenticated by STR profiling) in 2017 and 2024. Cells were cultivated at 37 °C and 5% CO_2_ and maintained in RPMI 1640 medium supplemented with 10% heat-inactivated fetal-bovine serum (Sigma Chemical Co., St. Louis, MO). The cultures were regularly tested for *Mycoplasma* contamination and experiments were conducted within 15 passages after authentication. Additional experiments were performed using RPMI media without glucose or l-glutamine (Gibco, Grand Island, New York, USA) under normoxic and hypoxic conditions using a c-chamber equipped with a ProOxC21O_2_/CO_2_ Controller (Biospherix NY, USA), respectively. Hypoxic conditions were predefined using 5% CO_2_/ 1% O_2_. Tissue sampling was conducted from rapid research autopsies at the National Koranyi Institute of Pulmonology (Budapest, Hungary) as recently described [19]. Primary tumors were collected within four hours after death and immediately snap-frozen in liquid nitrogen. Rapid autopsies were carried out in accordance with the Declaration of Helsinki. Written informed consent was obtained from all patients and approved by the National Hungarian Ethics Committee (BM/15090-1/2023).

### Flow cytometry

Cells (5 × 10^5^) were stained with 100 nM MitoTracker Red CMXROS (Molecular Probes, Eugene, Oregon, United States) or 1 nM Bodipy 493/503 (Thermo Scientific, Waltham, MA, USA) for 15 min at room temperature in the dark. After incubation, cells were analyzed by flow cytometry using a DxFlex Clinical Flow Cytometer (Beckman Coulter, Brea, California, USA) and signal intensity was determined. The mean intensity of each cell line was standardized to unstained controls and data was further analyzed in GraphPad Prism 8.0.

### JC-1 assay

Mitochondrial membrane potential was quantified using the JC-1 Mitochondrial Membrane Potential Assay Kit (ab113850, Abcam). Briefly, cells (2 × 10^4^) were seeded into a black-plate, clear-bottom 96-well plate. The following day, cells were treated with either 100 µM carbonyl cyanide-p-trifluoromethoxyphenylhydrazone (FCCP) or vehicle control (DMSO) for 4 h. JC-1 (10 µM) dye was then added to each well for 30 min at 37 °C. After several washing steps, JC-1 monomers and aggregates were measured at 535 nm and 590 nm, respectively, using a VarioScan Lux microplate reader (Thermo Scientific).

### Cell viability assays

Cells (5–7.5 × 10^3^) were seeded into 96-well F-bottom plates. After 24 h incubation, perhexiline (MedChemExpress, Monmouth Junction, NJ, USA), metformin (Glucophage, Merck, Darmstadt, Germany), IACS-010759 (MedChemExpress), UK5099 (MedChemExpress), BPTES (MedChemExpress), 2-Deoxy-D-glucose (MedChemExpress), oligomycin (MedChemExpress), or orlistat (MedChemExpress) was added in ascending doses as indicated. Inhibitors were incubated for 72 h at 37 °C prior to measurement. After a total of 96 h, growth assays were either developed with EZ4U substrate (measurement at 450 nm and 620 nm, Biomedica, Vienna, Austria) or quantified using SYBR green as previously described [20].

### Clonogenic assay

For long-term assessment of drug treatment, cells (2 × 10^3^) were seeded in 6-well plates and incubated overnight. Metformin (0.5 µM), IACS-010759 (10 nM) and oligomycin (10 nM) were added and plates were incubated for two weeks. Cells were fixed with 70% ethanol for 30 min and air dried. Staining with crystal violet was performed at room temperature for 2 h. Each well was washed multiple times with water and destained using a 2% SDS solution. Photometric measurement was performed at 562 nm.

### DNA isolation and qPCR

DNA was isolated using the AllPrep DNA/RNA/miRNA Universal Kit (Qiagen, Venlo, Netherland). Viable cells were seeded in 6-well plates (5 × 10^5^) and incubated overnight. DNA isolation was performed according to the manufacturer´s instructions and DNA content was measured using a nanodrop photospectrometer. The relative mitochondrial DNA copy number was determined via qPCR using the following primers: ND1_F, 5′-CCCTAAAACCCGCCACATCT-3′, ND1_R, 5′-GAGCGATGGTGAGAGCTAAGGT-3′; HGB_F, 5′-GTGCACCTGACTCCTGAGGAGA-3′, HGB_R, 5′-CCTTGATACCAACCTGCCCAG-3′, as described elsewhere [21]. qPCR was performed on a 7500 Fast Real-Time PCR System (Applied Biosystems, Waltham, MA, USA) using a Maxima SYBR green/ROX qPCR Master Mix (Thermo Scientific) [22]. Mitochondrial content was described as 2^−∆Ct^ between the mitochondrial ND1 and the nuclear HBG.

### Fluorescence microscopy

Cells (2 × 10^4^) were seeded in Ibitreat 8-well chamber slides (IBIDI, Gräfelfing, Germany) and incubated overnight. Cells were stained with 0.1 µM MitoTracker Red CMXROS (Molecular Probes) and 4′,6-diamidino-2-phenylindole (DAPI). Imaging was performed using an Olympus/Evident IXplore SpinSR spinning disk confocal microscope with Yokogawa disc (EVIDENT Corporation, Tokyo, Japan). A 100x objective together with a 3.2x magnification changer was used to yield a total magnification of 320x. The excitation of 561 nm was used to detect the red fluorophore followed by emission filtering using 617/73 nm. For enhanced super resolution performance, a SoRa spinning disc (Yokogawa Electric Corporation, Tokyo, Japan) was selected. Data acquisition was performed using the ORCA-Fusion Digital CMOS Camera C14440-20UP (HAMAMATSU PHOTONICS K.K. Tokyo, Japan). Automated imaging analysis was conducted using deep learning neuronal networking via the CellSens Dimension software (version 4.1) for SCLC cell lines. The neuronal network was trained manually on representative areas for subsequent automated imaging.

### Oil red staining

Intracellular fat compartments were stained using oil red (Sigma, St. Louis, MO, USA) in isopropanol. Cultured cells were counted and 2.5 × 10^4^ cells were used for cytospin preparation. Subsequently, slides were snap-frozen in liquid nitrogen and stored at -80 °C. Prior to staining, the slides were thawed following 20 min incubation with diluted oil red solution (60% isopropanol). Counterstaining was conducted for 5 min using Gill´s hematoxylin (Merck). Slides were scanned using a SCAN II (3DHistech, Budapest, Hungary) automated slidescanner for bright-field images and evaluated using ImageJ.

### Transmission electron microscopy (TEM)

For spheroid generation, cells were seeded in RPMI (10% FCS, 20% methylcellulose) in round bottom 96-well suspension plates (Sarstedt, Nümbrecht Germany). Spheroids were allowed to grow for 48 h prior to fixation and embedding. TEM was performed using a FEI Tecnai G2 20 equipped with a FEI Eagle 4 K CCD-Camera at the Electron Microscopy Facility in the Center for Anatomy and Cell Biology (Medical University of Vienna, Austria). Images were acquired using 80 kV and evaluated using ImageJ.

### Determination of cell size

Cells were harvested and placed on a cover slip. Pictures were taken with a Micro Ti Eclipse FL microscope, (Nikon, Minato City, Tokyo, Japan). At least 50 individual cells were measured with ImageJ and the volume calculated using the formula V = 4/3*r^3^*π.

### Seahorse assays

The cell lines DMS53 (12 × 10^3^) and H372 (6 × 10^3^) were seeded in Seahorse XFp Cell Culture Microplates to obtain 80% confluence and incubated overnight. The sensor cartridge was hydrated in calibrant and incubated at 37 °C in a non-CO_2_ incubator. On the next day, assay medium was prepared (1 mM pyruvate, 2 mM glutamine and 10 mM glucose) and added to each well after three washing steps. Inhibitor solutions were prepared to yield a final concentration of 1.5 µM oligomycin, 2 µM FCCP and 0.5 µM rotenone/antimycin A for the Seahorse XF Mito Stress Test and 3 µM BPTES, 4 µM etomoxir and 2 µM UK5099 for the Seahorse XF Mito Fuel Flex Test. Inhibitors were loaded into injection points of sensor cartridges and Hoechst 33258 Staining Dye Solution (Abcam, Cambridge, United Kingdom) was added to port four at a final concentration of 4 µM. Hoechst 33250 dye was used to normalize the data on cell count using Cytation and the Gen5 Software. Assays were performed and analyzed using the Wave Pro 10.1.0.1 software and the Seahorse XF Cell Mito Stress Test/ Mito Fuel Flex Test Report Generator.

### GSH/GSSG assay

Levels of glutathione (GSH) and glutathione disulfide (GSSG) were determined using the GSH/GSSG-Glo Assay (Promega, Wisconsin, USA) according the manufacturer’s instructions. Cells (2 × 10^4^) were seeded onto a white opaque 96-well plate and incubated overnight. Luminescence signals were measured using a VarioScan Lux microplate reader (Thermo Scientific).

### Enzyme activity assay

Cellular and tissue enzyme activity assays for succinate dehydrogenase (SDH), lactate dehydrogenase (LDH), isocitrate dehydrogenase (IDH), glutamate dehydrogenase (GDH), and 3 hydroxyacyl coenzyme A dehydrogenase (HAD) were performed based on previously reported enzymehistochemistry methods [23, 24]. In brief, 7 × 10^4^ cells were seeded onto round glass cover slips (Carl Roth) in a 24-well plate, incubated overnight, washed with 1x phosphate-buffered saline (PBS), and subsequently frozen at -80 °C. Activity assays were performed by thawing slides for 3 min at RT and then incubating sections with an enzyme specific reaction mixture in 0.1 M Tris-Maleate buffer at pH 8.5 for HAD, pH8 for GDH or pH 7.5 for LDH and IDH3 containing: 10% polyvinyl alcohol, 0.45 mM methoxy-phenanzine methosulfate, 5 mM sodium azide, 2 mM nitroblue tetrazolium chloride. The 0.1 M Tris-HCl buffer at pH 8 for mitochondrial SDH activities contained 0.2 mM phenanzine methosulfate instead of methoxyphenanzine methosulfate. To ensure specificity of the signals, we additionally included enzyme inhibitors sodium oxamate (100 mM) for LDH, oxaloacetic acid (100 mM) for IDH3, malonic acid (250 mM) for SDH, ATP (50.5 mM) for GDH and no substrate for HAD in the reaction mixtures as negative controls. Enzyme activity was developed for 15 min (LDH, HAD) and 20 min (SDH, IDH, GDH) for SCLC cell lines. The activity assays were stopped by washing with pre-warmed (60 °C) PBS and cells were stained with DAPI for nuclear cell segmentation and mounted with Fluoromount-G. Imaging and corresponding analysis was performed using an Olympus ix83 WF inverted microscope. The CellSens Dimension software (version 4.1) in combination with the “Deep learning” and “Count and Measure” packages were used. Automated object detection was conducted using neuronal network based on manual training and labeling. The single-cell enzyme activity values of 20 representative images were used for statistical analysis. Rapid autopsy samples were used for tissue-based analysis. Tumors were cut into 5 μm cryosections using a Cryostat Leica CM3050 and all five enzymes were analyzed. The development took 4 min for LDH and HAD, 15 min for IDH, 18 min for GDH, and 20 min for SDH. Images were again acquired using the Olympus ix83 WF inverted microscope and staining intensity was evaluated using the HALO software (Indica labs, Albuquerque, USA).

### Immunohistochemistry

Formalin-fixed, paraffin-embedded primary tumors obtained from rapid research autopsies were cut in 4 μm sections. ASCL1, NEUROD1, POU2F3, and YAP1 expressions were examined by immunohistochemistry (IHC). In brief, the sections were deparaffinized and heated in 10 mM citrate buffer (pH 6.0) in a pressure cooker for 20 min, except for NEUROD1 staining. These slides were heated in 10 mM Tris-EDTA (pH 9.0). Non-specific background staining was reduced using 0.3% H_2_O_2_ solution for 10 min. Antibody incubation was performed using anti-ASCL1 (BD Bioscience, San José, CA, #556604, 1:50), anti-NEUROD1 (Abcam, Cambridge, UK, #ab213725, 1:100), anti-POU2F3 (Santa Cruz Biotechnology, Dallas, TX, #sc-293402, 1:100), and anti-YAP1 (Cell Signaling Technology, Leiden, The Netherlands, #4912, 1:200) as described elsewhere [9, 25]. Antibody binding was visualized by using the ImmPACT DAB Substrate Kit (Vector Laboratories, Newark, California, United States) and nuclei were counterstained using hematoxylin.

### Proteomics and biostatistical analysis

**Fig. 1:**
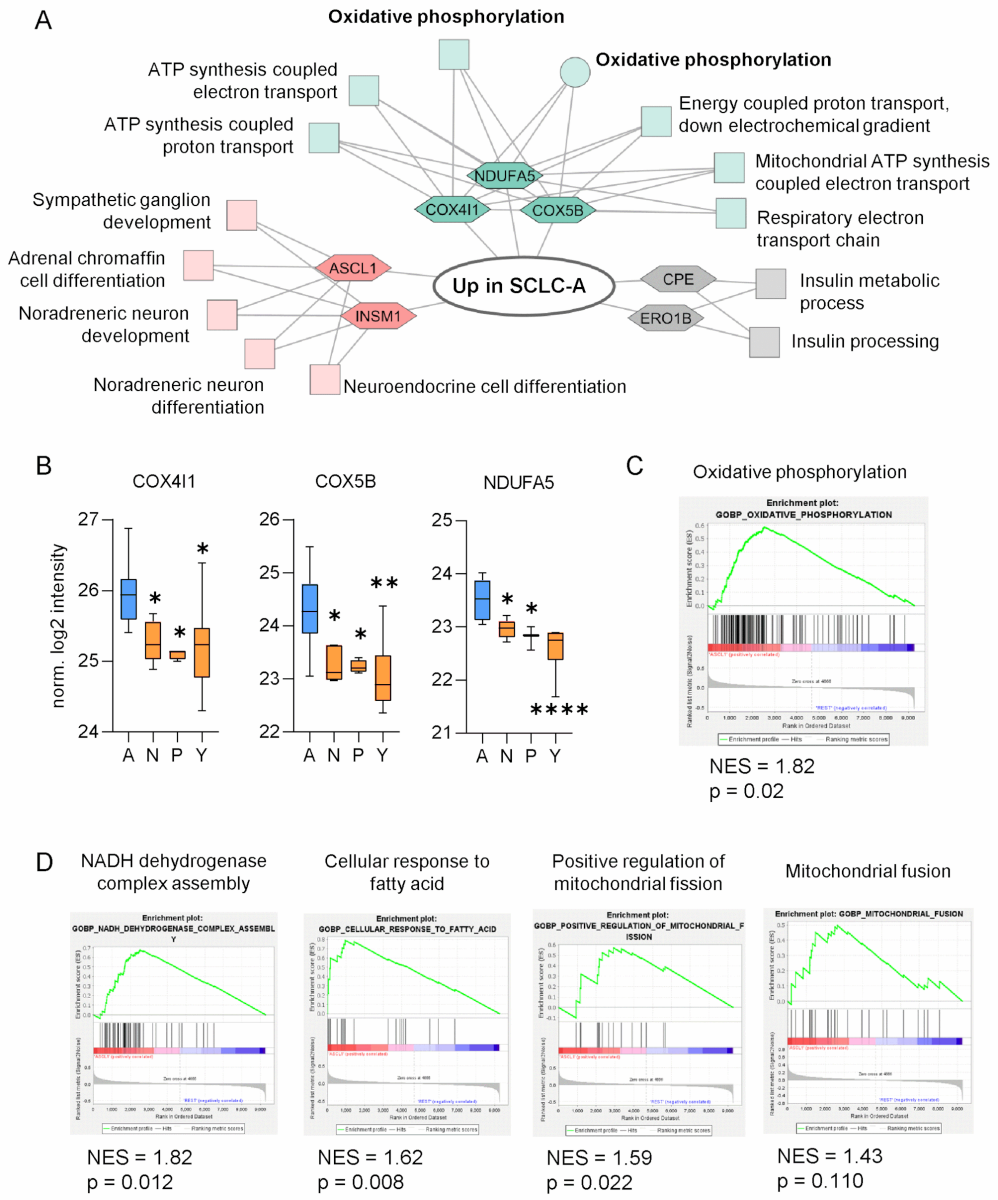
ASCL1 expression is associated with an oxidative phenotype in SCLC according to proteomic data. (**A**) ToppCluster analysis of significantly overexpressed proteins in the SCLC-A subtype (DMS153, DMS53, SHP77, H146, H1688, H1882, H209, H378) based on the comparison to non-SCLC-A cell lines (GLC4, H1694, H2171, H446, H524, H82, N417, COR-L311, H1048, H211, H526, CRL-2066, CRL-2177, H1341, H196, H372, H841, HLHE). The FDR cut-offs for KEGG (illustrated as a circle) and GOPB (shown as squares) pathways were 0.05 and 0.01, respectively. Hexagons imply the proteins related to the significant pathways. (**B**) Significantly overexpressed proteins COX4I1, COX5B and NDUFA5 in SCLC-A (blue) in SCLC-A cell lines compared to SCLC-N/P/Y (orange). Statistical significance was evaluated using the Kruskal-Wallis test. Data are represented as mean ± SEM. (**C**) Gene set enrichment analysis (GSEA) showing enriched oxidative phosphorylation in SCLC-A. (**D**) Differentially expressed pathways in SCLC-A resulting from GSEA. The y-axis indicates the normalized enrichment score (NES). * *p* ≤ 0.05, ** *p* < 0.01, *** *p* < 0.001, **** *p* < 0.0001

For the analyses shown in Fig. 1, we used our previously published proteomic dataset [18]. All details regarding sample preparation, mass spectrometry, and data processing are described in this publication. We re-analyzed the dataset by comparing SCLC-A (DMS153, DMS53, SHP77, H146, H1688, H1882, H209, H378) to all non-SCLC-A cell lines (GLC4, H1694, H2171, H446, H524, H82, N417, COR-L311, H1048, H211, H526, CRL-2066, CRL-2177, H1341, H196, H372, H841, HLHE). Gene set enrichment analysis (GSEA) was performed using the GSEA software version 4.3.2 [26, 27]. For further biostatistical analysis, t-tests between SCLC-A cell lines and non-SCLC-A cell lines (SCLC-N/P/Y) using the normalized log2 protein intensities were performed, and *p*-value < 0.05 was considered statistically significant. ToppCluster pathway analysis was conducted using the Cincinnati Children´s Biomedical Informatics Tool [28]. Databases that were considered for these analyses comprise KEGG (Kyoto Encyclopedia of Genes and Genomes) pathways, Gene ontology of biological processes, and the Hallmark gene set collection [29, 30, 31].

**Fig. 2:**
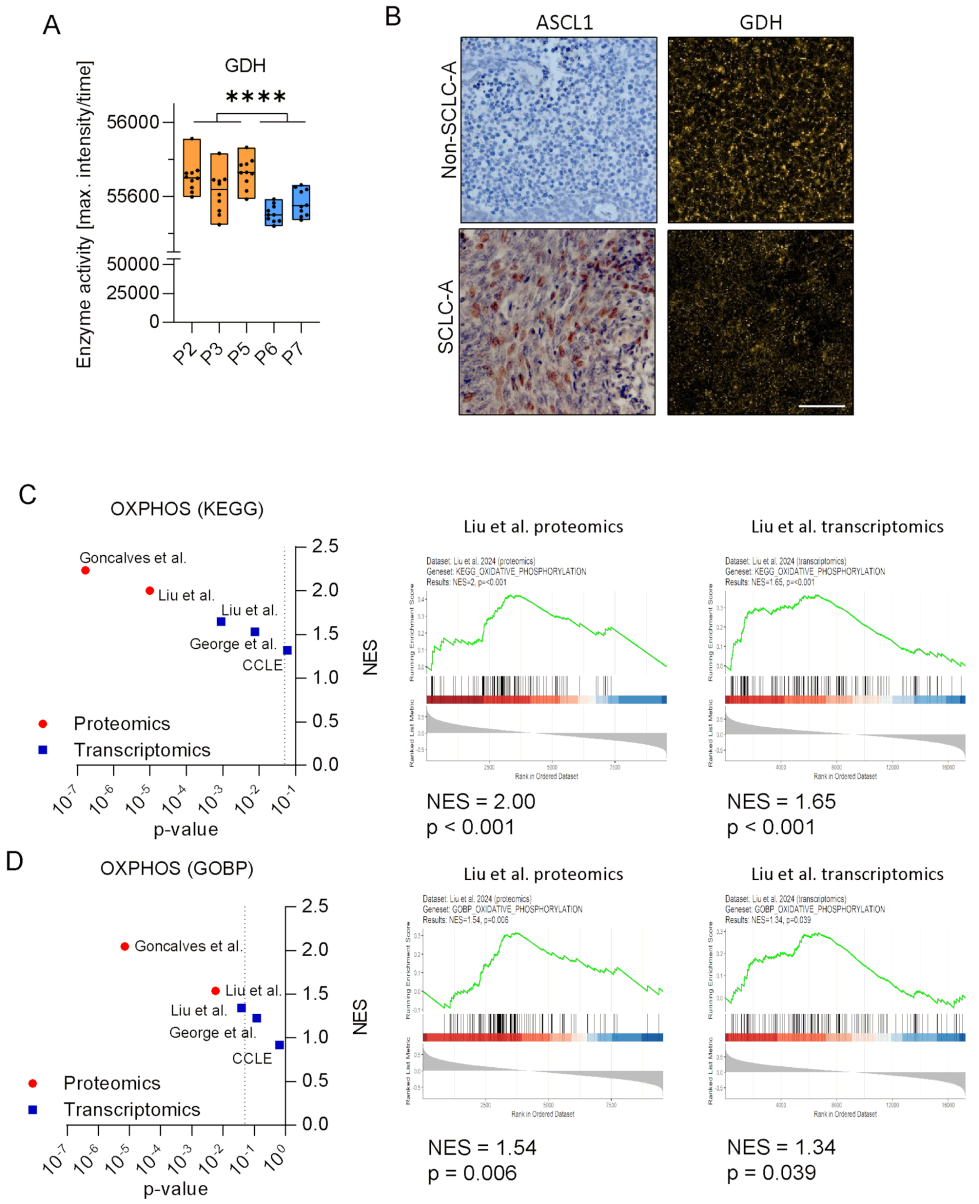
ASCL-1 expression correlates with reduced GDH activity and enrichment for oxidative phosphorylation in patient-derived tumor samples. (**A**) Average positive intensity of GDH in snap frozen primary tumors of SCLC patients. Orange and blue color indicate non-SCLC-A and SCLC-A-dominant phenotypes, respectively. (**B**) Corresponding representative images depicting ASCL1 expression (left) and GDH activity (right) in non-SCLC-A and SCLC-A-dominant primary tumors. Scale bar: 100 μm. Normalized enrichment scores (NES) and *p*-values of pre-ranked GSEA analyses of publicly available databases using the (**C**) KEGG and (**D**) GOBP geneset. The dotted lines indicate the treshold for statistical significance (*p* < 0.05). Representative GSEA plots from the Liu et al. dataset are shown

In Fig. 2, several publicly available databases for patent-derived proteomic and transcriptomic data were used. Due to the high prevalence of mixed subtypes in SCLC patients, pre-ranked GSEA was performed, with proteins/genes ranked based on their Pearson correlation against the ASCL1 gene expression. The analysis was performed using the GSEA function from clusterProfiler R package v4.12.6. Genesets from MSigDB v2024.1 were tested. GSEA plots were made with enrichplot R package v1.24.4. From the study of Liu et al. proteomic and transcriptomic data were used [32]. The 107 primary SCLC samples were annotated with their ASCL1 gene expression. The normalized protein and gene expression data were retrieved from Table S1 of the publication. Only proteins without missing expression values were kept (this step was not relevant for the transcriptomic data). To rank the proteins/genes for pre-ranked GSEA, the expression values were correlated with the samples’ corresponding ASCL1 gene expression using Pearson correlation. These correlation coefficients were used for ranking. Transcriptomic data from George et al. [7] was accessed from cBioPortal as previously described [33] The 78 primary and 3 metastatic SCLC samples were annotated with their ASCL1 gene expression (Z-score normalized expression values). Only rows without missing expression values were kept. Transcriptomic data from the Cancer Cell Line Encyclopedia (CCLE) was accessed and processed as previously described in Szeitz et al. [33] The 50 SCLC cell lines were annotated with their ASCL1 gene expression. The gene expression data were retrieved from the Cancer Dependency Map website and then processed to get the CPM-normalized data. To rank the genes for pre-ranked GSEA, for both datasets, the expression values were correlated with the samples’ corresponding ASCL1 gene expression using Pearson correlation. When multiple rows (transcripts) matched to the same gene, then their correlation values were averaged. These correlation coefficients were used for ranking of the genes. The proteomic data from Table 3 of Goncalves et al. [34] was accessed as normalized protein expression. Out of all SCLC cell lines, 36 were also part of the CCLE transcriptomic data, and those cell lines were annotated with their ASCL1 gene expression (CPM-normalized expression values). Only proteins without missing expression values were kept. To rank the proteins for pre-ranked GSEA, the expression values were correlated with the samples’ corresponding ASCL1 gene expression using Pearson correlation. Proteins without gene names were removed, and when multiple rows (proteins) matched to the same gene, then their correlation values were averaged. These correlation coefficients were used for ranking of the genes.

Statistical analysis for the respective figures was performed in GraphPad Prism 8. Unless stated otherwise, results are shown as mean ± SEM of at least three independent biological experiments. Differences were evaluated by Student’s T-test or ANOVA for comparisons of two or multiple groups, respectively, and considered statistically significant at *p* ≤ 0.05. (* *p* ≤ 0.05, ** *p* < 0.01, *** *p* < 0.001, **** *p* < 0.0001).

## Results

### The SCLC-A subtype is associated with higher oxidative phosphorylation and altered core metabolic processes

Previously published proteomic data on human SCLC cell lines revealed that the ASCL1-driven SCLC-A molecular subtype features a distinct metabolic phenotype, indicating a shift toward increased oxidative phosphorylation (OXPHOS) [18]. We now re-analyzed the proteome by grouping the cells into SCLC-A (*n* = 8) versus all non-SCLC-A subtypes together (*n* = 18), according to the predominant expression of the respective transcription factors ASCL1 (A), NEUROD1 (N), POU2F3 (P) and YAP1 (Y). Subsequent comprehensive ToppCluster analysis of proteins differentially expressed in SCLC-A vs. non-SCLC-A confirmed enrichment in OXPHOS and other core respiratory mechanisms, which highlights the unique metabolic nature of this SCLC subtype (Fig. 1A). According to a more detailed pairwise comparison, SCLC-A showed a significant upregulation of proteins Cytochrome C Oxidase Subunit 4 Isoform 1 (COX4I1), Cytochrome C Oxidase Subunit 5B (COX5B) and NADH Dehydrogenase (Ubiquinone) 1 Alpha Subcomplex 5 (NDUFA5) as compared to other (N/P/Y-expressing) subtypes, indicating increased mitochondrial activity (Fig. 1B). Notably, numerous other mitochondrial proteins involved in the electron transport chain and controlling mitochondrial dynamics were found to be dysregulated as illustrated in Supplementary Figures S1 and S2, but only NDUFA5, COX4I1 and COX5B were uniformly significantly upregulated within each pairwise subtype comparison.

To gather more information about the underlying cellular mechanisms with a focus on metabolic changes, we performed gene set enrichment analysis (GSEA) comparing all SCLC-A versus all non-SCLC-A cell lines. Intriguingly, more than half of the top 50 biological processes (ranked by the respective normalized enrichment score (NES)) were related to mitochondrial metabolism (Supplementary Table 1). Metabolic changes included significant enrichment in oxidative phosphorylation (NES = 1.82, *p* = 0.02), NADH dehydrogenase complex assembly (NES = 1.82, *p* = 0.012), cellular response to fatty acid (NES = 1.62, *p* = 0.008), and positive regulation of mitochondrial fission (NES = 1.59, *p* = 0.022). Additionally, mitochondrial fusion was enriched in SCLC-A cells, although not meeting the threshold for statistical significance (NES = 1.43, *p* = 0.11) (Fig. 1 C and D).

### SCLC-A is associated with higher mitochondrial content

To functionally assess the role of oxidative phosphorylation, we used a panel of six SCLC cell lines with the highest (DMS53, SHP77, H1688; further termed OXPHOS^high^) and lowest (H841, H372, H196; OXPHOS^low^) protein expressions of NDUFA5, COX4I1, and COX5B (Supplementary Figure S3). Of note, all OXPHOS^high^ cell lines were ASCL1-driven, whereas OXPHOS^low^ cell lines were exclusively YAP1 positive.

**Fig. 3:**
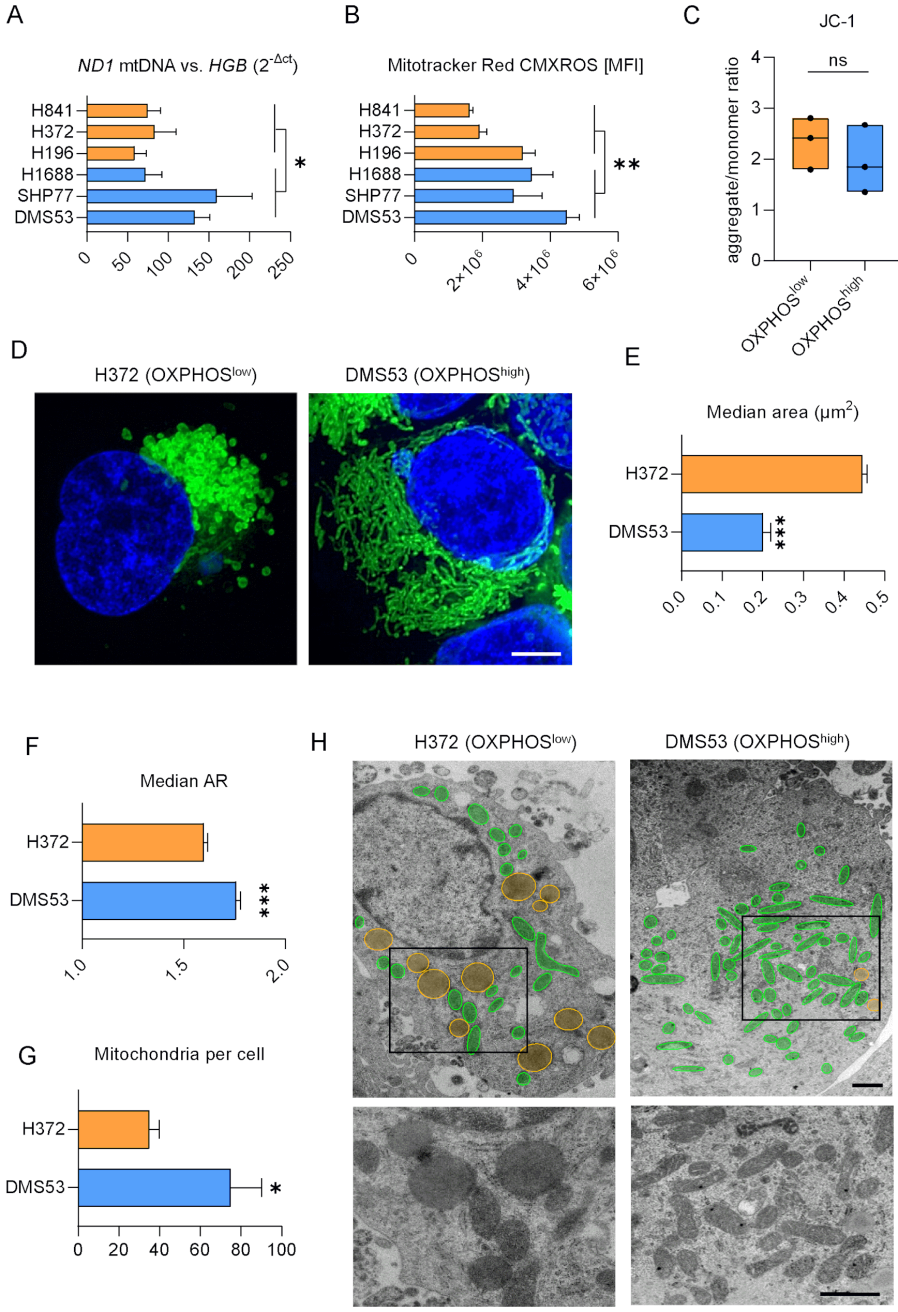
OXPHOS^high^ cells have altered mitochondrial composition. (**A**) Mitochondrial content was determined via mitochondrial *ND1.* Nuclear *HGB* expression was used as reference gene. The results indicate significantly more mitochondria in OXPHOS^high^ cell lines (DMS53, H1688 and SHP77) compared to OXPHOS^low^ (H196, H372, H841). RNA levels were measured by qPCR and results are shown as 2^−Δct^. Statistical significance was determined using the Mann-Whitney test (*p* = 0.0242). (**B**) Fluorescent staining of mitochondria was measured using the mean fluorescence intensity (MFI) of Mitotracker Red CXMROS via flow cytometry and normalized to unstained controls (Mann-Whitney test; *p* = 0.0075). (**C**) Ratio of JC-1 aggregates and monomers in OXPHOS^high^ (blue) and OXPHOS^low^ (orange) cell lines. Each dot represents the mean of one cell line. T-Test, ns: not significant (*p* = 0.477). (**D**) Representative images of mitochondria stained with Mitotracker Red CMXROS of a cell line with low (left, H372) and high (right, DMS53) oxidative characteristics. Scale bar: 5 µM. (**E**) Median area and (**F**) median aspect ratio (AR) of mitochondria in the OXPHOS^low^ H372 and the OXPHOS^high^ DMS53 cell lines were determined using automated image analysis of *n* > 10^5^ individual mitochondria (Mann-Whitney test). Mitochondrial size and shape were evaluated from confocal images using neuronal AI network and showed smaller mitochondrial areas and increased AR of DMS53 indicating an elongated shape compared to H372. (**G**) Significantly higher mean number of mitochondria was determined in DMS53 (OXPHOS^high^) compared to H372 (OXPHOS^low^) based on transmission electron microscopy images (*n* = 5, respectively). Mann-Whitney tests * *p* ≤ 0.05, ** *p* < 0.01, *** *p* < 0.001. Data are represented as mean ± SEM. (**H**) Images of transmission electron microscopy of mitochondria from the OXPHOS^low^ cell line H372 (left panel) and the OXPHOS^high^ cell line DMS53 (right panel). Upper images illustrate mitochondria in green and lipid droplets in yellow. Middle images show overviews of representative images and bottom images depict higher magnification excerpts. Scale bar: 1 μm

Quantification of the mitochondrial DNA copy number using qPCR revealed a significantly higher (*p* = 0.0242, mean fold change: 1.75) mitochondrial content in the OXPHOS^high^ group. This was determined by the difference between the mitochondrial NADH dehydrogenase 1 (*ND1*) and the nuclear hemoglobin gene (*HGB*) (Fig. 3A). Flow cytometry analysis in the respective cell lines following staining with Mitotracker Red CMXROS confirmed a higher number of mitochondria in the OXPHOS^high^ group (*p* = 0.0021, mean fold change: 1.74). A JC-1 assay, which enables to assess mitochondrial membrane potential, revealed no significant difference (*p* = 0.477) in membrane potential between OXPHOS^high^ and OXPHOS^low^ cells, as shown by the ratio of JC-1 aggregates and monomers (Fig. 3C, Supplementary Figure S4A). Of note, when we treated the cells with an uncoupler of mitochondrial oxidative phosphorylation (FCCP), as expected, we found a reduction of JC1 aggregates (Supplementary Figure S4B). Confocal imaging of a representative OXPHOS^high^ (DMS53) and OXPHOS^low^ (H372) cell line stained with Mitotracker Red CMXROS also demonstrated a greater abundance of mitochondria in DMS53 (Fig. 3C, Supplementary Figure S5).

### OXPHOS^high^ and OXPHOS^low^ cells differ in mitochondrial size and shape

Interestingly, we observed remarkable differences in mitochondrial shape and size between OXPHOS^high^ and OXPHOS^low^ cell lines. While the OXPHOS^high^ cell line DMS53 showed elongated mitochondria, H372 displayed round mitochondrial enlargement, indicating alterations in mitochondrial function [35] (Fig. 3D, Supplementary Figure S5). We used a deep learning neuronal network for automated image analysis of *N* > 10^5^ individual mitochondria to quantify the observed differences in size and shape. Accordingly, while H372 showed a significantly greater median mitochondrial area and sphericity, the mitochondria of DMS53 were characterized by significantly higher median aspect ratio (AR = proportional relation between width and height), proportion of mitochondria with AR > 2, and median elongation factor (EF) per image (*N* = 8 per cell line) analyzed (Fig. 3E and F, Supplementary Figure S6). In-depth structural analysis using TEM validated higher mitochondrial content in DMS53 compared to H372 based on significantly higher mitochondrial numbers (Fig. 3G and H). Moreover, the mean area of mitochondria as well as the circularity were significantly lower in DMS53, whereas the mean AR was lower in H372 (Supplementary Figure S7), which is in concordance with our above results (Fig. 3E and F).

### OXPHOS^high^ cells are smaller and exhibit less intracellular lipid storage

**Fig. 4:**
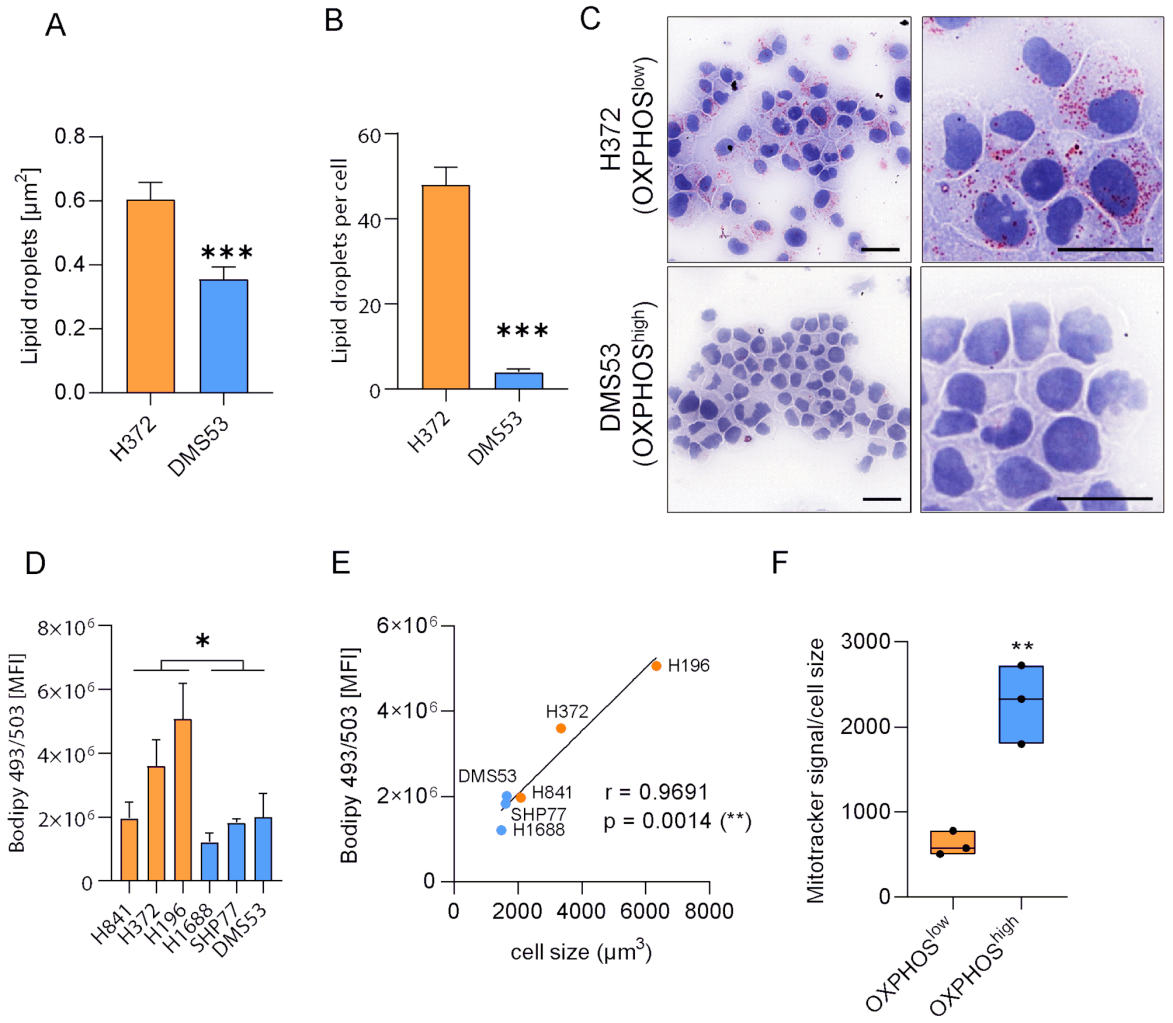
OXPHOS^high^ cell lines are smaller and exhibit less intracellular lipid storage. (**A**) Evaluation of electron microscopy showing a significantly increased area (µm^2^) of lipid droplets in OXPHOS^low^ (H372) and OXPHOS^high^ (DMS53) cell lines. (**B**) Lipid droplets were stained with oil red, manually counted and compared between H372 (orange bar) and DMS53 (blue bar) cells confirming the observed differenced in lipid storage. (**C**) Oil red staining of lipid droplets of two representative cell lines. Scale bar: 20 μm. (**D**) Lipid compartments in the OXPHOS^high^ (DMS53, H1688 and SHP77) and the OXPHOS^low^ cell lines (H196, H372, H841) were stained with Bodipy 493/503 and the mean fluorescence intensity (MFI) was measured via flow cytometry. Mann-Whitney tests * *p* ≤ 0.05, ** *p* < 0.01, *** *p* < 0.001. Data are represented as mean ± SEM. (**E**) Pearson correlation between cell size (x-axis) and cellular lipid content (y-axis) in OXPHOS^high^ (blue) and OXPHOS^low^ (orange) cell lines. Each dot represents the mean of one cell line. (**F**) Mitochondrial content measured by Mitotracker Red CXMROS (see Fig. 2B) normalized to cell size. Each dot represents the mean of one OXPHOS^high^ (blue) or OXPHOS^low^ (orange) cell line. T-Test. ** *p* < 0.01

Notably, TEM revealed significantly larger lipid droplets in the OXPHOS^low^ cell line H372, indicating increased storage of intracellular lipids (Figs. 3H and 4A, Supplementary Figures S7D and E). Quantitative evaluation of oil red stainings in H372 and DMS53 cells confirmed a significant lipid accumulation in OXPHOS^low^ cells and depleted lipid levels in OXPHOS^high^ cells (Fig. 4B and C). Since fatty acids represent an important energy source for ATP generation via oxidative phosphorylation, we further investigated intracellular lipid droplets in our panel of six cell lines. Flow cytometry following Bodipy 495/503 lipid staining revealed a significantly higher overall content of lipid droplets in OXPHOS^low^ cells (*p* = 0.018, mean fold change: 2.14; Fig. 4D). Furthermore, we found a high cellular size variation in the OXPHOS^low^ cell lines (vs. OXPHOS^high^ cells with minimal size variation and a statistically not significant smaller size; *p* = 0.10; Supplementary Figure S8A). When we compared cell size to lipid storage, we found a highly significant correlation (*p* = 0.0014, *r* = 0.9691, Fig. 4E), resulting in no difference when intracellular lipid content was normalized to cell size (*p* = 0.485, Supplementary Figure S8B). Importantly, the mitochondria content in relation to cell size remained significantly different between OXPHOS^high^ and OXPHOS^low^ cells (*p* = 0.0039), further strengthening our findings that OXPHOS^high^ cells have a higher number and also density of mitochondria (Fig. 4F, Supplementary Figure S8C).

### OXPHOS activity dictates the reliance on lipid or glutamine metabolism

**Fig. 5:**
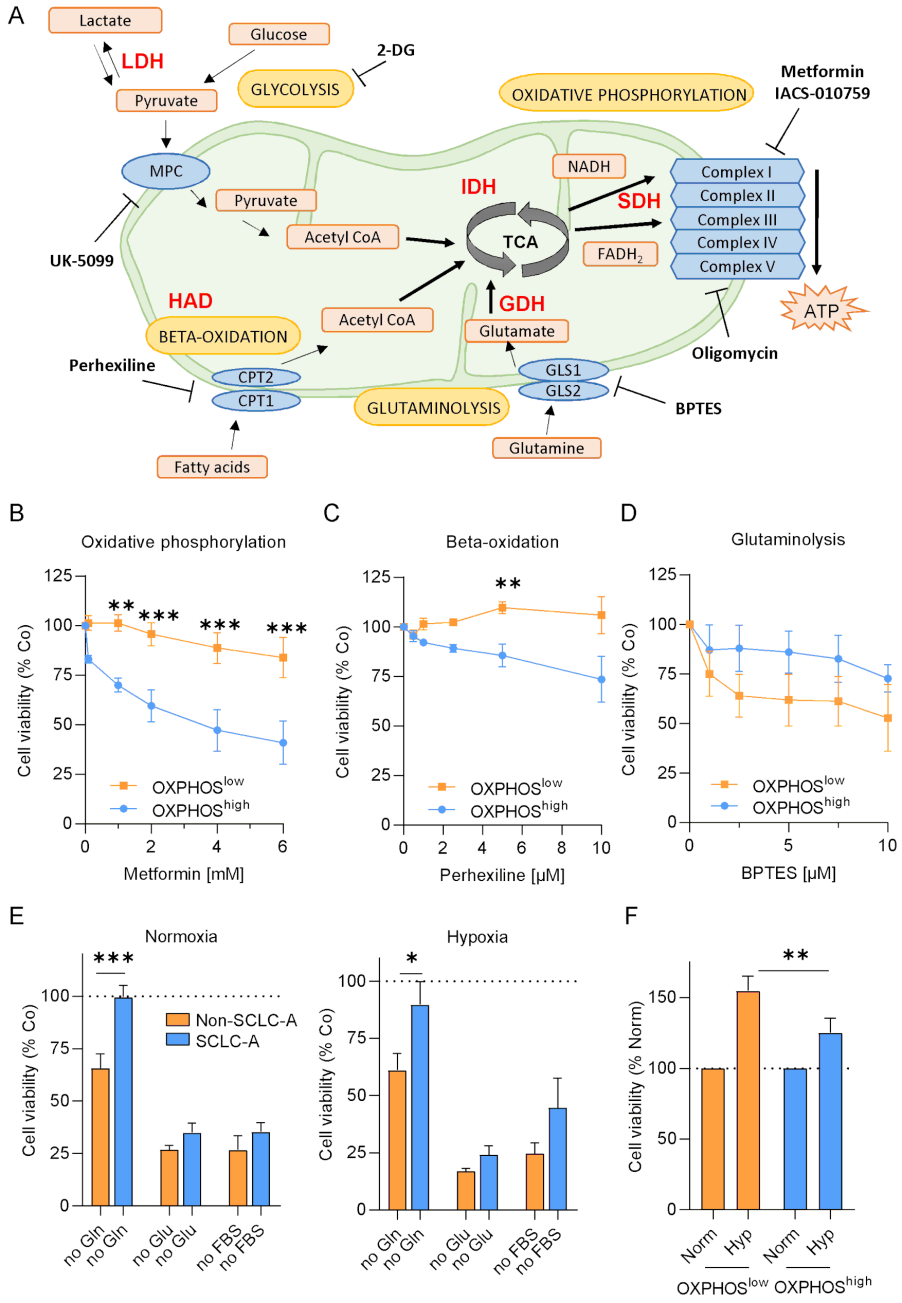
Screening of metabolic inhibitors reveals differential responses between cell lines with OXPHOS^high^ and OXPHOS^low^ signatures. (**A**) Illustration of all tested inhibitors and their corresponding targets. Enzymes investigated via activity assays are shown in red (GDH– glutamate dehydrogenase, IDH - isocitrate dehydrogenase, SDH-succinate dehydrogenase, LDH– lactate dehydrogenase and HAD − 3 hydroxyacyl coenzyme A dehydrogenase). Cell viability of three OXPHOS^high^ (blue) and OXPHOS^low^ (orange) cell lines after 72 h using increasing doses of (**B**) metformin, (**C**) perhexiline and (**D**) BPTES. Statistical significance was determined using 2-way ANOVA and Sidak´s multiple comparisons test. (**E**) Growth assays using modified media without glucose, glutamine or FBS were performed under normoxic (left) and hypoxic (right) conditions over 72 h in OXPHOS^high^ (blue) or OXPHOS^low^ (orange) cell panels (N per subgroup = 3, respectively). (**F**) Comparison of cell growth behavior between hypoxic and normoxic conditions OXPHOS^high^ (blue) or OXPHOS^low^ (orange) cell panels. Mann-Whitney tests. * *p* ≤ 0.05, ** *p* < 0.01, *** *p* < 0.001, **** *p* < 0.0001. Data are represented as mean ± SEM

Next, we screened for therapeutic vulnerabilities using various metabolic inhibitors (Fig. 5A). First, to validate the dependency on oxidative phosphorylation, we treated cells with the widely used mitochondrial respiratory complex I inhibitor metformin and the small molecule inhibitor IACS-010795, as well as with the ATP synthase (Complex V) inhibitor oligomycin (Fig. 5A). Pooled results of cell viability assays indicated greater effects in the three OXPHOS^high^ (compared to the three OXPHOS^low^ cell lines) with all inhibitors (Fig. 5B, Supplementary Figure S9). In support of this, in colony formation assays we observed much more pronounced effects using smaller doses of each inhibitor in the representative OXPHOS^high^ cell line SHP77 compared to the OXPHOS^low^ cell line H196 (Supplementary Figure S10).

To elucidate the reliance on preceding metabolic pathways fueling the tricarboxylic acid cycle (TCA) including glycolysis, β-oxidation, and glutaminolysis, cells were also treated with the mitochondrial pyruvate carrier (MPC) inhibitor UK-5099, the competitive glycolysis inhibitor 2-deoxy-D-glucose (2-DG), the carnitine palmitoyltransferase 1 (CPT1) inhibitor perhexiline, and the glutaminase (GLS) inhibitor BPTES, targeting different metabolic routes (Fig. 5A). While all cell lines were equally resistant to glycolysis inhibition, OXPHOS^high^ cells showed hypersensitivity to perhexiline (β-oxidation inhibition) while cell lines with a OXPHOS^low^ profile were more vulnerable to glutaminolysis inhibition using BPTES (Fig. 5 C and D, Supplementary Figure S11), suggesting that OXPHOS^high^ cells rather depend on lipids to maintain their high oxidative state. This is in line with the observed lower amount of lipids in OXPHOS^high^ cell lines (Fig. 4). To exclude sensitivity patterns based on differential levels of targets, respective protein expression patterns of the different SCLC subtypes were evaluated in all 26 cell lines using our proteomic data. Except for MPC2 expression, which was higher in ASCL-1 expressing compared to NEUROD1 and YAP1-dominant cells, protein intensities were similarly distributed between SCLC-A and the other molecular subtypes (Supplementary Figure S12).

Next, we tested the reliance on glutamine, glucose, or fetal bovine serum by incubating the cells with modified media under normoxic and hypoxic conditions over 72 h. Confirming our previous results, cell growth was only significantly inhibited in the OXPHOS^low^ cells when glutamine was absent. Intriguingly, this phenomenon was detected under both normoxic and hypoxic conditions (Fig. 5E, Supplementary Figures S13A and B). Regarding glucose and serum starvation, cell viability was generally low with no difference between OXPHOS^high^ and OXPHOS^low^ cells, regardless of the presence of oxygen (Fig. 5E, Supplementary Figures S13A and B). Of note, hypoxia over 72 h generally triggered proliferation in all cell lines tested (Fig. 5F, Supplementary Figure S13C).

### OXPHOS^high^ cells are more aerobic and depend on fatty acid synthesis

Next, we aimed to further confirm our results in Seahorse assays. Indeed, the OXPHOS^high^ DMS53 and the OXPHOS^low^ H372 cell lines showed significant variances in oxygen consumption rate (OCR) and extracellular consumption rate (ECAR) (Supplementary Figures S14A-C). Looking at the baseline levels of OCR and ECAR of the Seahorse Mito Stress Test, representative OXPHOS^high^ cells displayed different metabolic states. Hence, DMS53 cells were characterized as aerobic cells, whereas H372 cells were particularly associated with glycolytic functions (Supplementary Figure S14D). Overall, OXPHOS^high^ cells depicted significantly higher ATP production (Supplementary Figure S14E). Assessment of the metabolic dependency and capacity based on the Seahorse Mito Fuel Flex Test further confirmed these results (Supplementary Figures S14F and G). Dependency, in this respect, defines the reliance of SCLC cells on appointed metabolites by the cell´s inability to compensate for absent fuels. Both representative cell lines were most dependent on glucose consumption. However, significantly higher dependency of glutamine was observed in the OXPHOS^low^ H372 cell line whereas dependency on fatty acids for ATP production was significantly higher in OXPHOS^high^ DMS53 cells (Supplementary Figure S14F). Metabolic capacity indicates the ability of maintaining baseline respiration of a certain fuel pathway when the other two metabolites are diminished. Glucose and fatty acid capacities were significantly more pronounced with slightly more than 70% and 30% in the ASCL1-dominant DMS53 cells, respectively (Supplementary Figure S14G). Of note, glutamine capacity was comparable.

### OXPHOS^low^ cells display increased glutamate dehydrogenase activity and glutathione levels

**Fig. 6:**
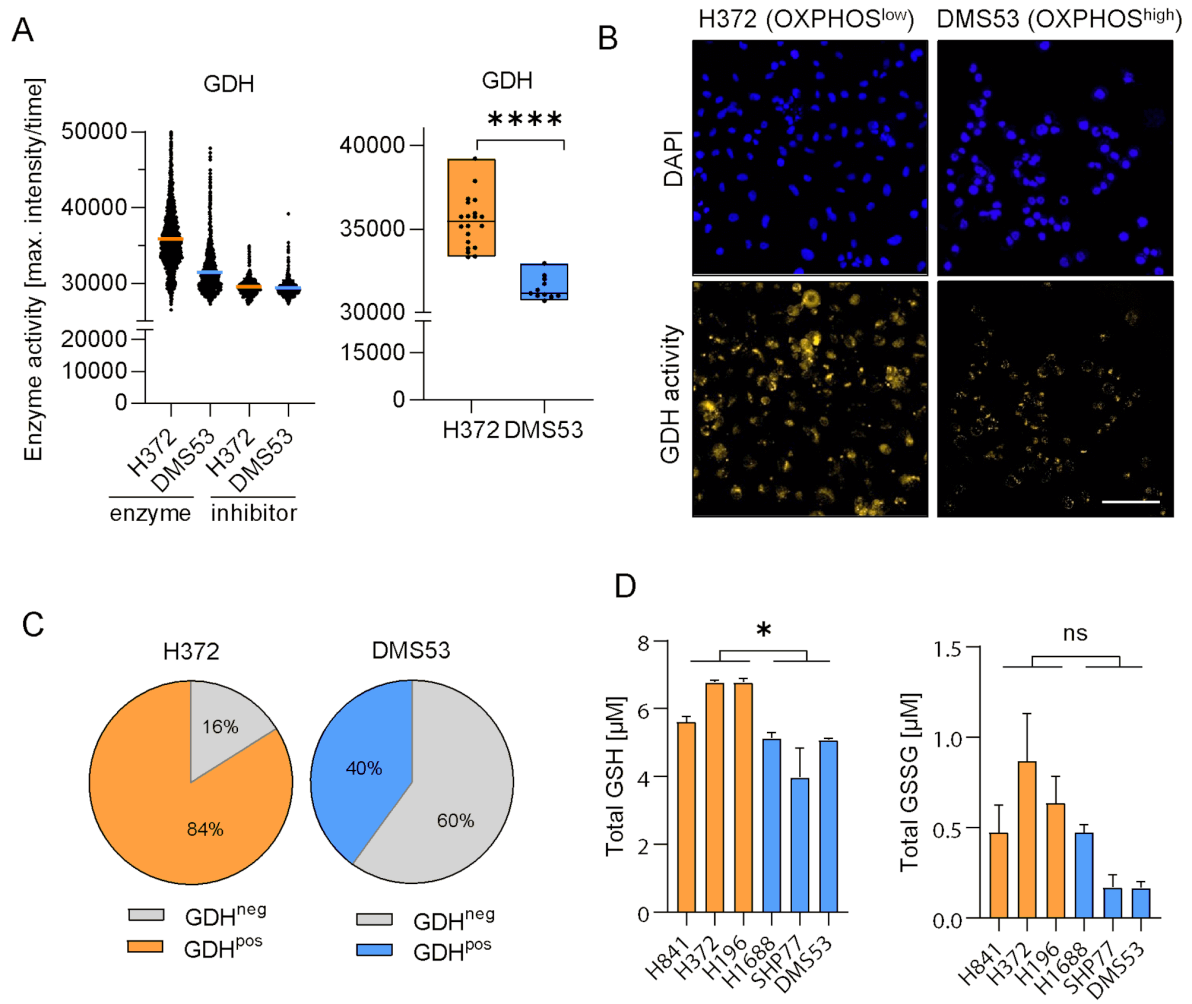
OXPHOS^low^ cells exhibit increased GDH enzyme activity. (**A**) GDH activity in the OXPHOS^low^ cell line H372 (orange) compared to the OXPHOS^high^ cell line DMS53 (blue) on single-cell level with and without the inhibitor ATP (left) and max. enzyme activity per image (*n* = 20, respectively; right). Statistical significance was determined using the Mann-Whitney test. **** *p* ≤ 0.0001. (**B**) Corresponding representative images depicting nuclei (blue) and GDH activity (yellow) in H372 (right) and DMS53 (left) cells. Scale bar: 50 μm. (**C**) Frequency distribution (cut-off defined by respective negative inhibitor staining, 97.3%) in DMS53 (blue) and H372 (orange) cells. (**D**) Total glutathione (GSH) and glutathione disulfide (GSSG) levels in OXPHOS^high^ cell lines (DMS53, H1688 and SHP77) compared to OXPHOS^low^ (H196, H372, H841). Statistical significance was determined using the Mann-Whitney test. * *p* ≤ 0.05. Data is shown as mean ± SEM

In order to determine the activity of important metabolic enzymes such as lactate dehydrogenase (LDH), succinate dehydrogenase (SDH), isocitrate dehydrogenase (IDH), glutamate dehydrogenase (GDH), and 3-hydroxyacyl coenzyme A dehydrogenase (HAD), we performed enzyme activity assays as described previously [23]. We observed significantly higher enzymatic activity (*p* < 0.0001, mean fold change: 1.13) of GDH in the OXPHOS^low^ H372 compared to DMS53 cells (Fig. 6A and B), further supporting their dependency on glutaminolysis. Frequency distribution analysis categorizing the cells in subpopulations with negative or positive stainings (cutoff defined by respective negative controls) revealed 84% GDH positivity in H372 compared to 40% in DMS53 (Fig. 6C). GDH activity inside the nuclei was equally distributed between both cell lines (Supplementary Figure S15). Likewise, IDH activity displayed a similar activity pattern and frequency distribution in these representative SCLC cells being more active in OXPHOS^low^ cells. Taking a closer look at ASCL1-driven DMS53 cells, the activity levels of HAD, SDH and LDH were higher as compared to H372 cells (Supplementary Figures S15-19). Further analysis of the nuclei revealed augmented enzyme activities of HAD and SDH, but especially LDH in the SCLC-A cells, whereas IDH did not show differential nuclear distributions (Supplementary Figures S15-19). Of note, we found slightly higher glutathione (GSH) levels in the OXPHOS^low^ cell lines (*p* = 0.036, mean fold change: 1.35), while glutathione disulfide (GSSG) levels, although trending in the same direction, were not significantly (*p* = 0.0644) different (Fig. 6D).

Finally, we set out to validate our findings in patient-derived samples, using human SCLC tissue specimens and publicly available datasets. First, we conducted enzyme activity assays in human primary tumor tissues obtained from rapid research autopsies of five SCLC patients. Pathological evaluation of immunohistochemical stainings for ASCL1 revealed that two specimens were from the SCLC-A subtype (P6 and P7), while the remaining three (P2, P3 and P5) were largely negative for ASCL1 and thus represented non-SCLC-A tumors (Supplementary Figure S20). While SDH, LDH, and HAD showed no significantly different activity, GDH and IDH levels were significantly higher in non-SCLC-A tumors, which is in line with our in vitro findings (Fig. 2A and B, Supplementary Figure S21).

Furthermore, to validate the importance of oxidative phosphorylation in SCLC-A patient-derived data, we analyzed a several publicly available proteomic and transcriptomic datasets. Specifically, pre-ranked GSEA was performed using the KEGG and GOBP genesets for oxidative phosphorylation on proteomic data from Liu et al. [32] and transcriptomic data from Liu et al. [32] and George et al. [7]. In addition, we analyzed two cell line datasets, proteomics from Goncalves et al. [36] and transcriptomics from the CCLE. Our data show a significant (*p* < 0.05, indicated by the dotted lines) enrichment of OXPHOS in SCLC-A in all datasets except for the cell line data from CCLE and the GOBP data from George et al. (Figs. 2 C and D, Supplementary Figure S22). Generally, we found higher normalized enrichment scores in the proteomic data compared to transcriptomic data, indicating a more accurate representation of cellular metabolism at the protein level (Fig. 2 C and D).

## Discussion

Although novel therapeutic approaches including immune checkpoint inhibition have extended the therapeutic options for SCLC, the clinical outcome of SCLC patients remains poor. Major contributors to negative clinical trial endpoints in SCLC may be high tumor plasticity as well as inadequate patient stratification based on underlying molecular backgrounds [8]. Hence, the investigation of SCLC subtypes and corresponding molecular profiles is fundamental for the development of diagnostic biomarkers and personalized therapies. Accordingly, we previously published an in-depth proteomic analysis of 26 human SCLC cell lines focusing on four subtypes based on the dominant expression of ASCL1, NEUROD1, POU2F3, and YAP1 [18]. In that study, we found that increased expression of the transcriptional regulator ASCL1 was associated with a metabolic shift towards oxidative phosphorylation [18].

ASCL1 is a transcription factor known for its critical role in regulating neuronal development. In the context of SCLC, ASCL1 has been described to mediate Wnt signaling, thereby influencing NE differentiation, tumor cell proliferation and promoting epithelial-like characteristics through E-cadherin expression [37]. Thus, SCLC is metabolically heterogeneous and driven by different levels of the lineage oncogene ASCL1 as evidenced by metabolomic results [38]. By analyzing the topmost enriched KEGG pathways and biological processes based on proteins differentially expressed in SCLC-A vs. non-SCLC-A, we identified increased NE differentiation and oxidative phosphorylation, but also altered insulin metabolism, signifying the NE origin and metabolic distinctiveness of ASCL1-driven SCLCs. Recently, oxidative metabolism has been described in various types of malignant tumors including melanoma or pancreatic carcinoma, among others [39, 40]. A distinct cluster of diffuse large B-cell lymphomas were related to increased oxidative phosphorylation in 2005, suggesting a differential metabolism in lymphoma cells [41]. Therefore, high oxidative phosphorylation activity may display a key regulatory mechanism of tumor metabolic reprogramming, especially in ASCL1-driven SCLC.

Next, we evaluated SCLC-A subtype-specific features using GSEA and found that several mitochondria-related processes were significantly enriched including NADH dehydrogenase complex assembly. Aberrations in complex I of the electron transport chain has been thoroughly investigated in health and disease, and represents the most frequent defect in aerobic respiration in human disorders [42]. In our current study, we demonstrate significantly upregulated expression levels of mitochondrial *ND1*. ND1 is located in the NADH dehydrogenase complex [42], hence, supporting our hypothesis of higher mitochondrial content and activity in SCLC-A cell lines. Moreover, overexpression of complex I has been found to be associated with higher metastatic potential in human breast cancer [43]. Intriguingly, we recently reported that high ASCL1 expression is associated with worse overall survival compared to other SCLC molecular subtypes in surgically-treated patients [9, 13]. Thus, high activity of complex I of the electron transport chain indicates a higher propensity of aggressiveness in cancer.

Mitochondrial fusion and fission are highly dynamic processes that play pivotal roles in preserving functional mitochondria [44]. In this study, both biological processes were dysregulated between SCLC-A and non-SCLC-A cell lines according to GSEA. Recently, SCLC has been associated with upregulation of commonly known transcription factors including ASCL1, ID2/4, or FOXA2, all potentially mediating mitochondrial processes such as mitochondrial organization and elongation [45]. In accordance with this finding, Gil and colleagues have recently reported pathological overrepresentation of oxidative phosphorylation and enrichment of processes required for mitochondrial translation in melanoma samples [46]. The balance between fusion and fission can be mediated by metabolic and pathogenic cellular environments, thereby influencing the maintenance of functional mitochondria, redistribution, and cell growth [47]. Recent in vitro data suggest that induction of mitochondrial fission promotes oxidative phosphorylation, whereas the inhibition of mitochondrial fission results in diminished oxidative phosphorylation in hepatic stellate cells [48]. However, similar to our observation in non-ASCL1 SCLC cells, mitochondrial dysfunction has been found to be associated with elevated expression of epithelial-to-mesenchymal transition (EMT) genes [49]. Indeed, by analyzing proteomic and/or transcriptomic data, we previously demonstrated strong associations of EMT-scores with POU2F3 expression in SCLC cells and, furthermore, showed that YAP1-dominant SCLC cells are related to EMT pathways (KEGG) [18].

Oxidative phosphorylation in mitochondria utilizes electrons from NADH and FADH2, which can be generated from the metabolism of long-chain fatty acids, glucose, and amino acids, to produce ATP. CPT-1 is located in the outer mitochondrial membrane, mediates the transport of long-chain fatty acids from the cytoplasm into the mitochondria that can further be metabolized via fatty acid β-oxidation [50]. Indeed, growing evidence suggests that β-oxidation and CPTs are fundamental for cell proliferation and viability, and also for promoting drug resistance in cancer [51]. Notably, our SCLC-A cohort was more sensitive to CPT1 inhibition by perhexiline, suggesting fatty-acids as oxidizable substrates metabolized by SCLC-A cells. Given the highly aggressive behavior of SCLC-A compared to the other SCLC subtypes [9], targeting the CPT system and lipid homeostasis may become an emerging target for SCLC-A.

Metformin has been used for the first-line treatment of type II diabetes mellitus (T2DM) by decreasing hepatic gluconeogenesis [52]. Recent evidence supports the cytotoxic activity of metformin via mTOR-pathway activation and inhibition of the complex 1 of the respiratory chain in mitochondria [53]. Nevertheless, the antineoplastic effects and efficacy of metformin in addition to chemotherapy in SCLC remains unclear. A recent meta-analysis evaluated six independent studies including more than 500 SCLC patients with T2DM who received concurrent metformin therapy [54]. These authors observed significantly longer overall and disease-free survivals in SCLC patients receiving metformin. Based on this information in addition to our findings, we hypothesize that the combination of metformin and cytotoxic therapy may increase the therapeutic efficacy primarily in ASCL1-expressing SCLCs.

We found significantly higher GDH activity in our non-SCLC-A cell lines and, accordingly, GDH expressions in non-SCLC-A human tumor samples obtained from rapid autopsies. Glutamine is one of the most abundant amino acids in the human body and its conversion to glutamate via glutaminase is essential for fueling the TCA cycle [55]. Glutamate serves a dual role in cellular processes. Firstly, it acts as a primary supplier of α-ketoglutarate (α-KG), playing a crucial role in both energy production and biosynthesis. Secondly, glutamate serves as a precursor for the essential cellular antioxidant glutathione, contributing to the maintenance of intracellular redox homeostasis [56]. In line with this, we found increased glutathione levels in the non-SCLC-A cell lines. Besides the glycolytic shift that proliferating cancer cells commonly acquire, they additionally display a dependence on other metabolites including glutamine [55]. Of note, glutamine metabolism has been described to be mediated in a MYC-dependent manner in different malignancies including SCLC [57]. MYC-high SCLC, associated with ASCL1-low SCLC subtypes, has additionally been shown to be more glycolytic with less oxidative capacity, hence, in accordance with our findings [58, 59]. Only recently, targeting the host glutamine metabolism has been demonstrated to result in increased chemosensitivity in SCLC mouse xenograft models [60]. The higher GDH activity in non-SCLC-A primary material again highlights different underlying metabolic dynamics that might aid in developing personalized therapeutic approaches in SCLC.

The better understanding of metabolic rewiring in SCLC offers new insights to this hard-to-treat disease. Our findings indicate that human ASCL1-dominant SCLCs primarily depend on high mitochondrial content and metabolic processes including oxidative phosphorylation to ensure their energy requirements. Based on this study, we propose two subtype-specific metabolic vulnerabilities in SCLC. Firstly, ASCL1-dominant SCLCs are more susceptible to complex I inhibition along with CPT1 inhibition. Secondly, non-ASCL1 dependent SCLCs are more vulnerable to glutamine depletion and inhibition of glutaminolysis. Our observations of varying GDH activities were also confirmed in human SCLC tissues, indicating similar enzymatic activities during disease progression. The importance of oxidative phosphorylation was also validated in patient-derived, publicly available datasets. Nevertheless, further studies are warranted to validate the susceptibility of SCLC-A tumors to OXPHOS inhibition and the superior response of non-SCLC-A tumors to glutaminolysis inhibition.

## Electronic supplementary material

Below is the link to the electronic supplementary material.

Supplementary Material 1

Supplementary Material 3

## Data Availability

No datasets were generated or analysed during the current study.

## Abbreviations

2-DG: 2-deoxy-D-glucose
α-KG: α-ketoglutarate
ART: Aspect ratio
ASCL1: Achaete-scute homologue 1
CCLE: Cancer Cell Line Encyclopedia
COX4I1: Cytochrome C Oxidase Subunit 4 Isoform 1
COX5B: Cytochrome C Oxidase Subunit 5B
CPT1: Carnitine palmitoyltransferase 1
DAPI: 4′,6-diamidino-2-phenylindole
ECAR: Extracellular consumption rate
EF: Elongation factor
EMT: Epithelial-to-mesenchymal transition
GDH: Glutamate dehydrogenase
GLS: Glutaminase
GSEA: Gene set enrichment analysis
GSH: Glutathione
GSSG: Glutathione disulfide
HAD: 3 hydroxyacyl coenzyme A dehydrogenase
HGB: Hemoglobin
ICI: Immune checkpoint inhibitor
IDH: Isocitrate dehydrogenase
IHC: Immunohistochemistry
LDH: Lactate dehydrogenase
MPC: Mitochondrial pyruvate carrier
NADH: Nicotinamide adenine dinucleotide
ND1: NADH dehydrogenase 1
NDUFA5: NADH dehydrogenase (ubiquinone) 1 alpha subcomplex 5
NE: Neuroendocrine
NES: Normalized enrichment score
NEUROD1: Neurogenic differentiation factor 1
OCR: Oxygen consumption rate
OXPHOS: Oxidative phosphorylation
PBS: Phosphate-buffered saline
POU2F3: POU class 2 homeobox 3
SCLC: Small cell lung cancer
SCLC-AN: ASCL1/ NEUROD1 expressing SCLC
SDH: Succinate dehydrogenase
TCA: Tricarboxylic acid cycle
TRX: Thioredoxin
T2DM: Type II diabetes mellitus
YAP1: Yes-associated protein 1
