# Supplementary material for: Unveiling the powerhouse: ASCL1-driven small cell lung cancer is characterized by higher numbers of mitochondria and enhanced oxidative phosphorylation
Source: Cancer Metab. 2025 Mar 31;13:16. doi: 10.1186/s40170-025-00382-6 (PMC11959836; doi:10.1186/s40170-025-00382-6)
Supplement: Supplementary file 2 — Supplementary Material 3 [file 40170_2025_382_MOESM2_ESM.pdf]

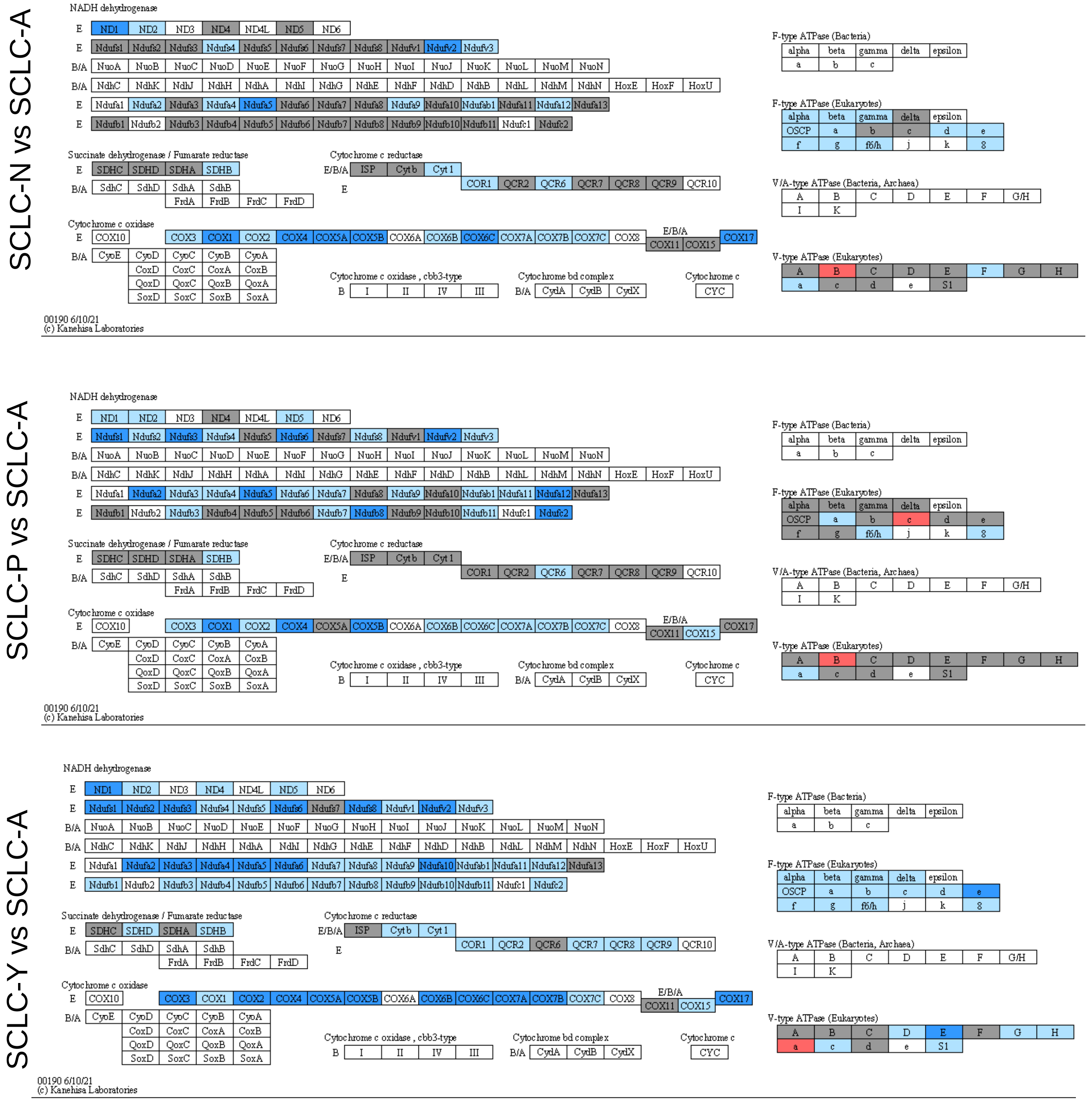

Supplementary Figure S1: Custom coloured KEGG pathway of oxidative phosporylation using the results from pairwise proteomic comparison between the SCLC subtypes. SCLC-N (top), SCLC-P (middle), and SCLC-Y (bottom) versus SCLC-A, repectively. Dark blue indicates significant downregulation in SCLC-N/P/Y, hence upregulation in SCLC-A (ANOVA p-value + Tukey Posthoc p<0.05), light blue highlights downregulated proteins in SCLC-N/P/Y and light red shows upregulated proteins in SCLC-N/P/Y (log2 FC > 0.5, regardless of the p-value).

Solta et al. Supplementary Figure S1

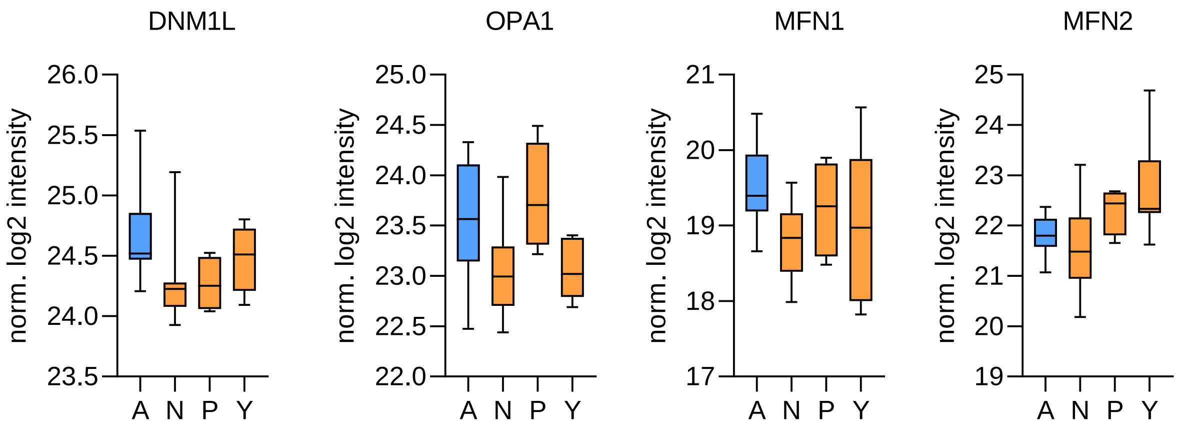

Supplementary Figure S2: Protein expression in SCLC subtypes. Expression of DNM1L, OPA1, MFN1 and MFN2 in SCLC-A (blue) compared to SCLC-N/P/Y (orange) cell lines according to proteomic data.

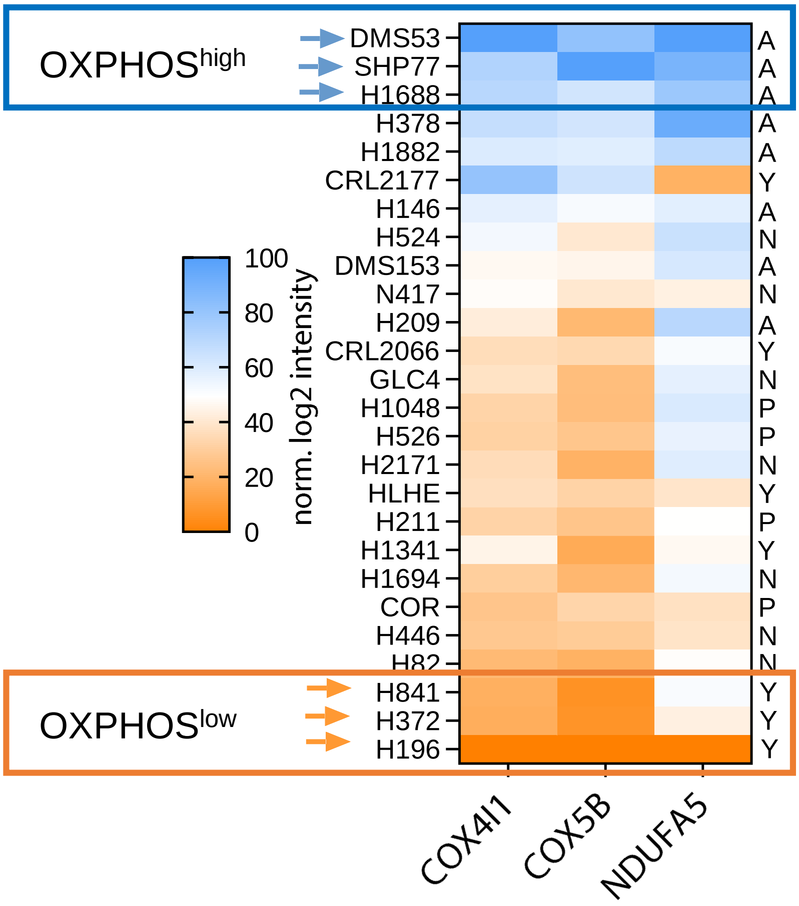

Supplementary Figure S3: Heatmap depicting the panel of SCLC cell lines associated with high and low oxidative phosphorylation activity. LFQ values obtained from proteomic analysis were normalized within each collumn to determine the topmost (blue) and bottommost (orange) SCLC cell lines with highest and lowest expression levels of COX4I1, COX5B and NDUFA5 to determine the OXPHOS^{high} and OXPHOS^{low} groups. Corresponding SCLC subtypes are listed on the right side of the panel (A: SCLC-A, P: SCLC-P, N: SCLC-N, Y: SCLC-Y).

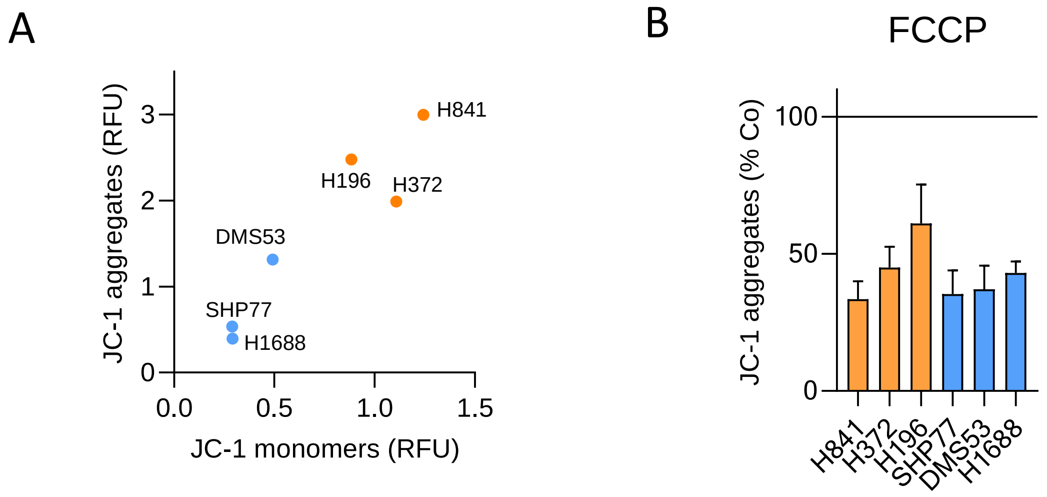

Supplementary Figure S4: Determination of membrane potential. (A) JC1 monomers and aggregates in OXPHOS^{high} (blue) and OXPHOS^{low} (orange) cell lines. (B) Amount of JC-1 aggregates after 4 h exposure to the uncoupler FCCP, normalized to untreatred control. Data is shown as mean +SEM.

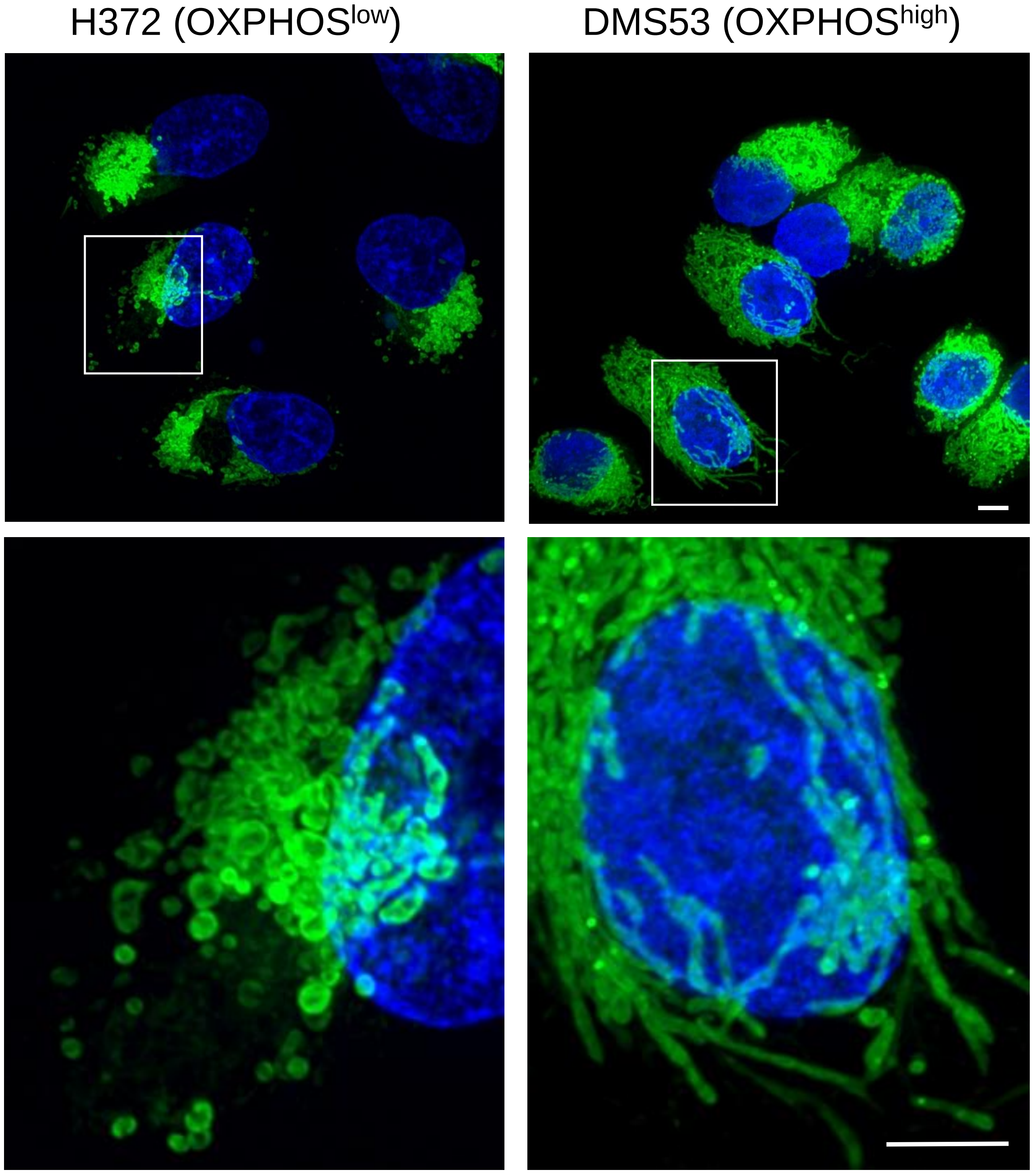

Supplementary Figure S5: Additional images of fluorescence stainings comparing H372 and DMS53. Structures indicated by green and blue represent mitochondria (Mitotracker CMXROS) and nuclei (DAPI), respectively. The lower images show higher magnification of the cells. Scale bar: 10 µm

Solta et al. Supplementary Figure S5

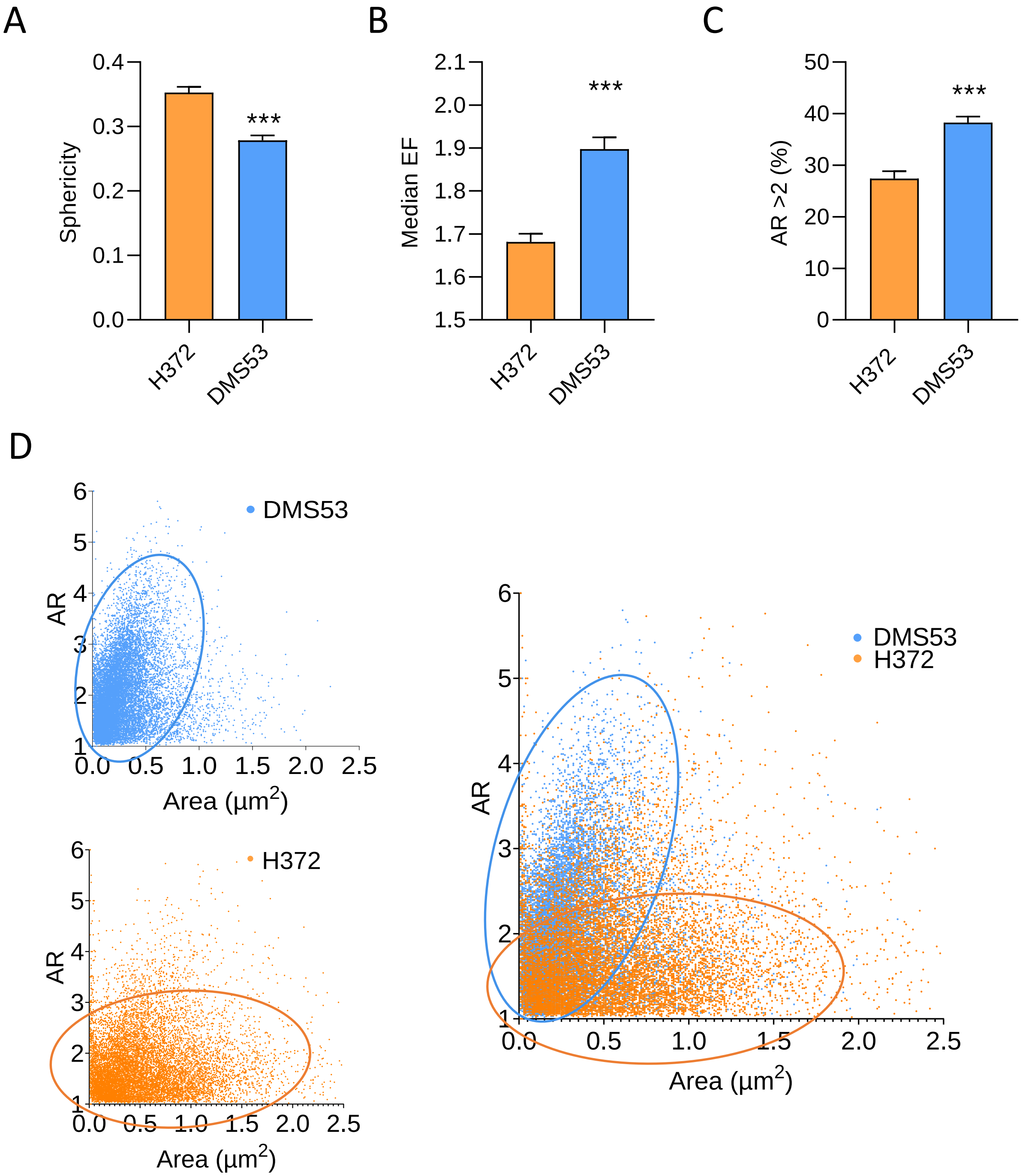

Supplementary Figure S6. Cells characterized by diffential oxidative signatures show variances in mitochondrial size and shape. (A) Sphericity (%), (B) median elongation factor and (C) aspect ratio >2 were determined using automated image analysis of n>105 individual mitochondria per cell line (H372 = OXPHOS^{low} = orange, DMS53 = OXPHOS^{high} = blue). Mann-Whitney tests. *** p<0.001. Data are represented as mean ± SEM. (D) Scatter plot of aspect ratio versus area representing every mitochondria measured between the two cell lines.

Solta et al. Supplementary Figure S6

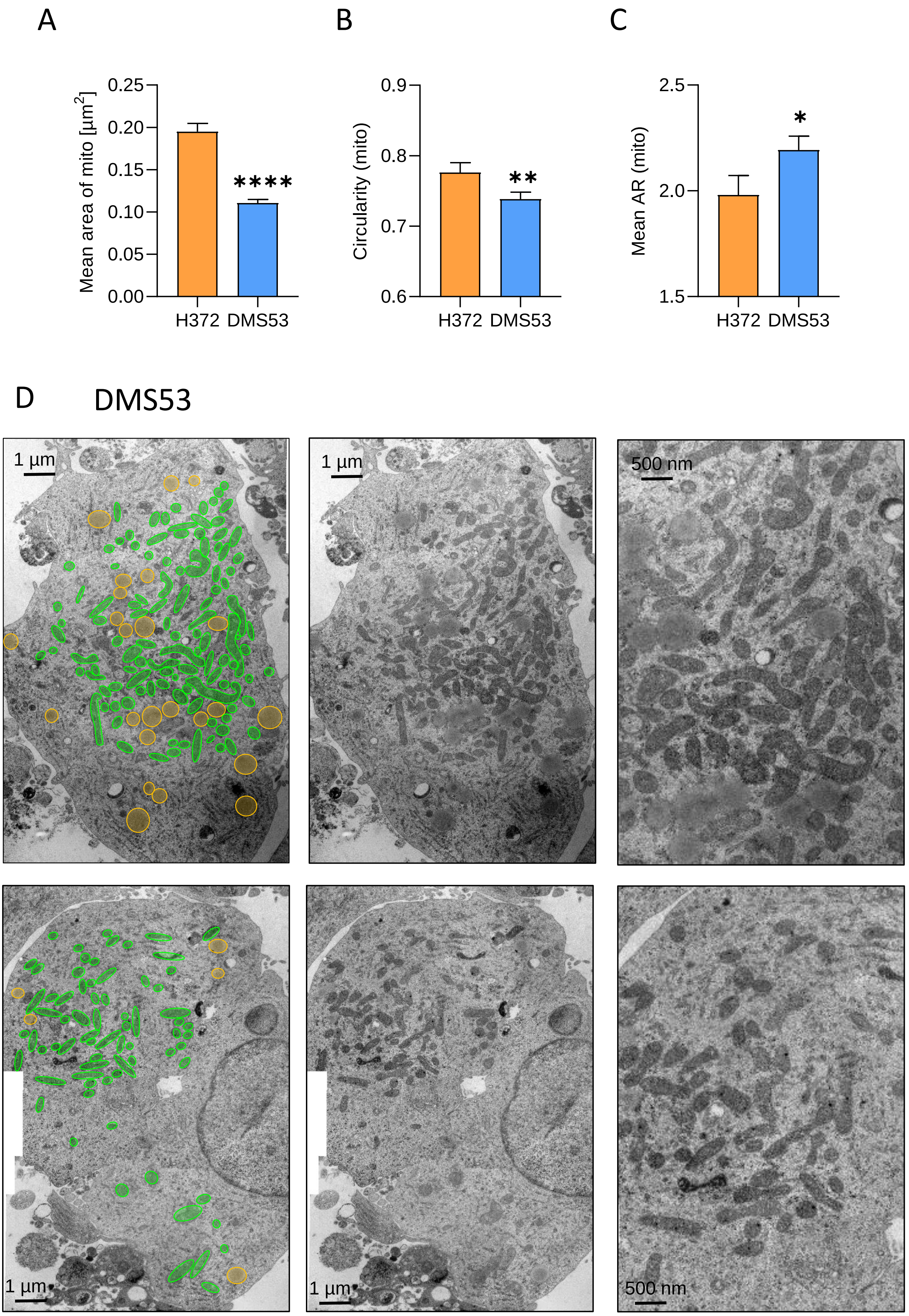

Solta et al. Supplementary Figure S7

E H372

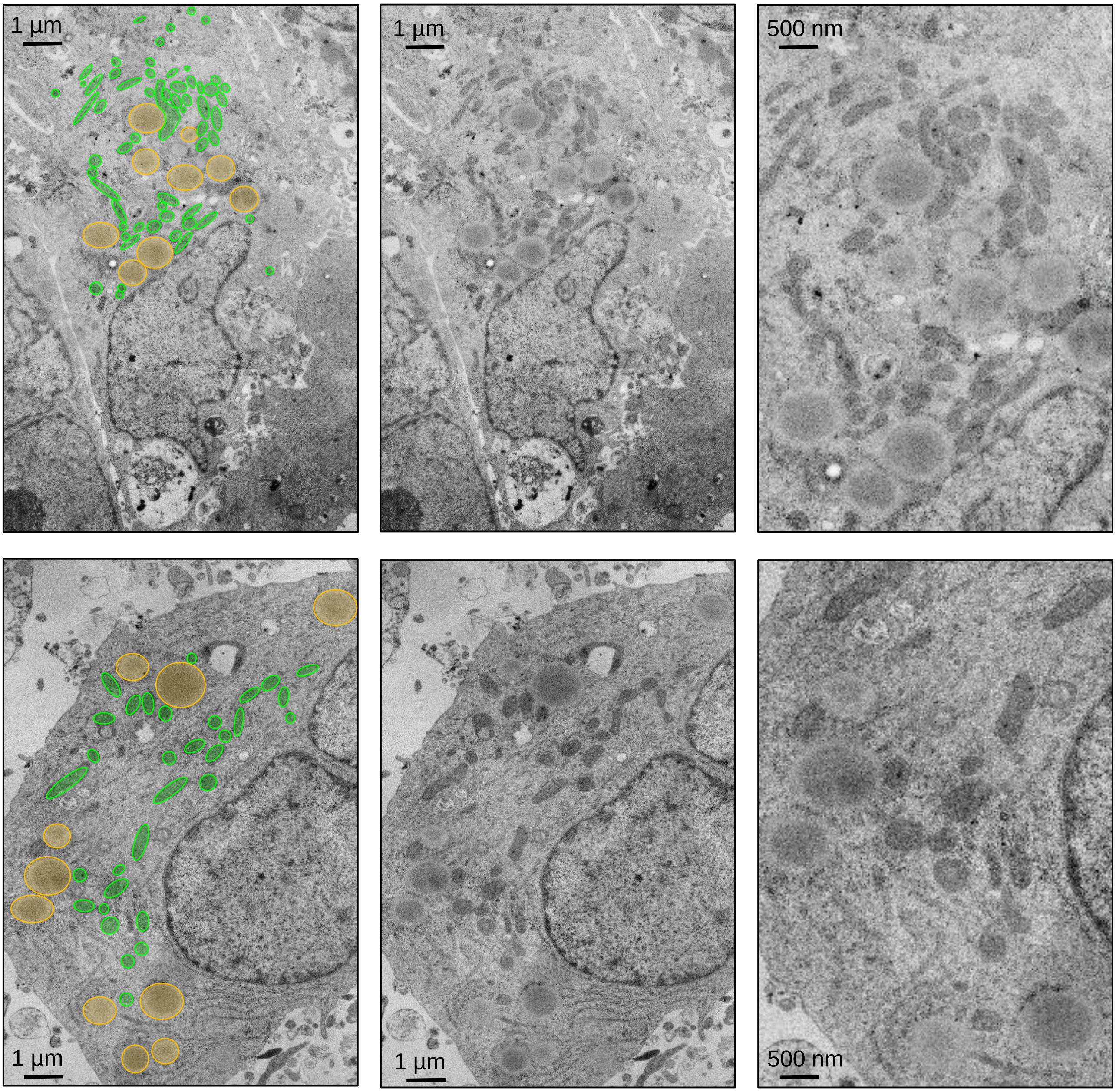

Supplementary Figure S7. Quantitative and structural assessment of mitochondria and lipid droplets using automated imaging or transmission electron microscopy independently validated higher mitochondrial content in DMS53 cells and more lipid droplets in H372 cells. (A) Mean mitochondrial area (µm^2), (B) mitochondrial circularity and (C) mean aspect ratio of mitochondria (images acquired from electron microscopy) were evaluated using ImageJ (H372 = OXPHOS^{low} = orange, DMS53 = OXPHOS^{high} = blue). Mann-Whitney tests. * $p < 0.05$, ** $p < 0.01$, *** $p < 0.001$, **** $p < 0.0001$. Data are represented as mean ± SEM. In-depth analysis of mitochondria in (D) DMS53 and (E) H372 using transmission electron microscopy. Green structures define mitochondria and yellow contours show lipid droplets.

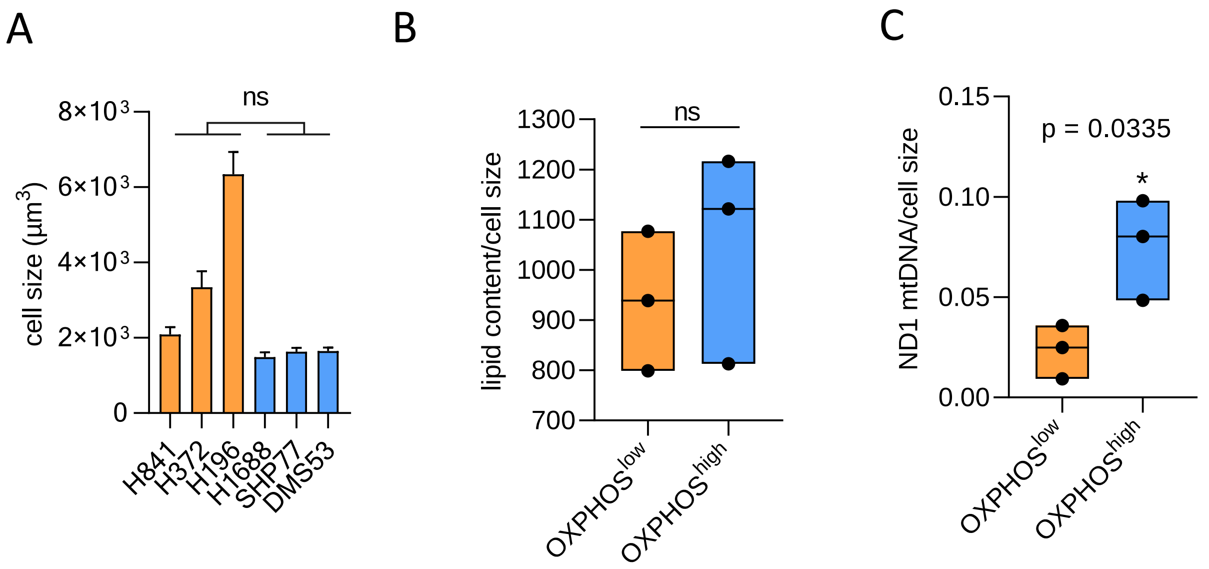

Supplementary Figure S8. Cell size in relation to lipid content and mitochondrial DNA. (A) Cell volume of three OXPHOS^{high} (blue) and OXPHOS^{low} (orange) cell lines determined by ImageJ. Data is shown as mean ± SEM of at least 50 individual cells per cell line. Mann-Whitney test. ns: non significant. (B) Cellular lipid content determined by Bodipy and (C) ND1 mtDNA assessed by qPCR relative to cell size in OXPHOS^{high} (blue) and OXPHOS^{low} (orange) cells. Each dot represents the mean of one cell line. T-test, * $p < 0.05$, ns: not significant.

Solta et al. Supplementary Figure S8

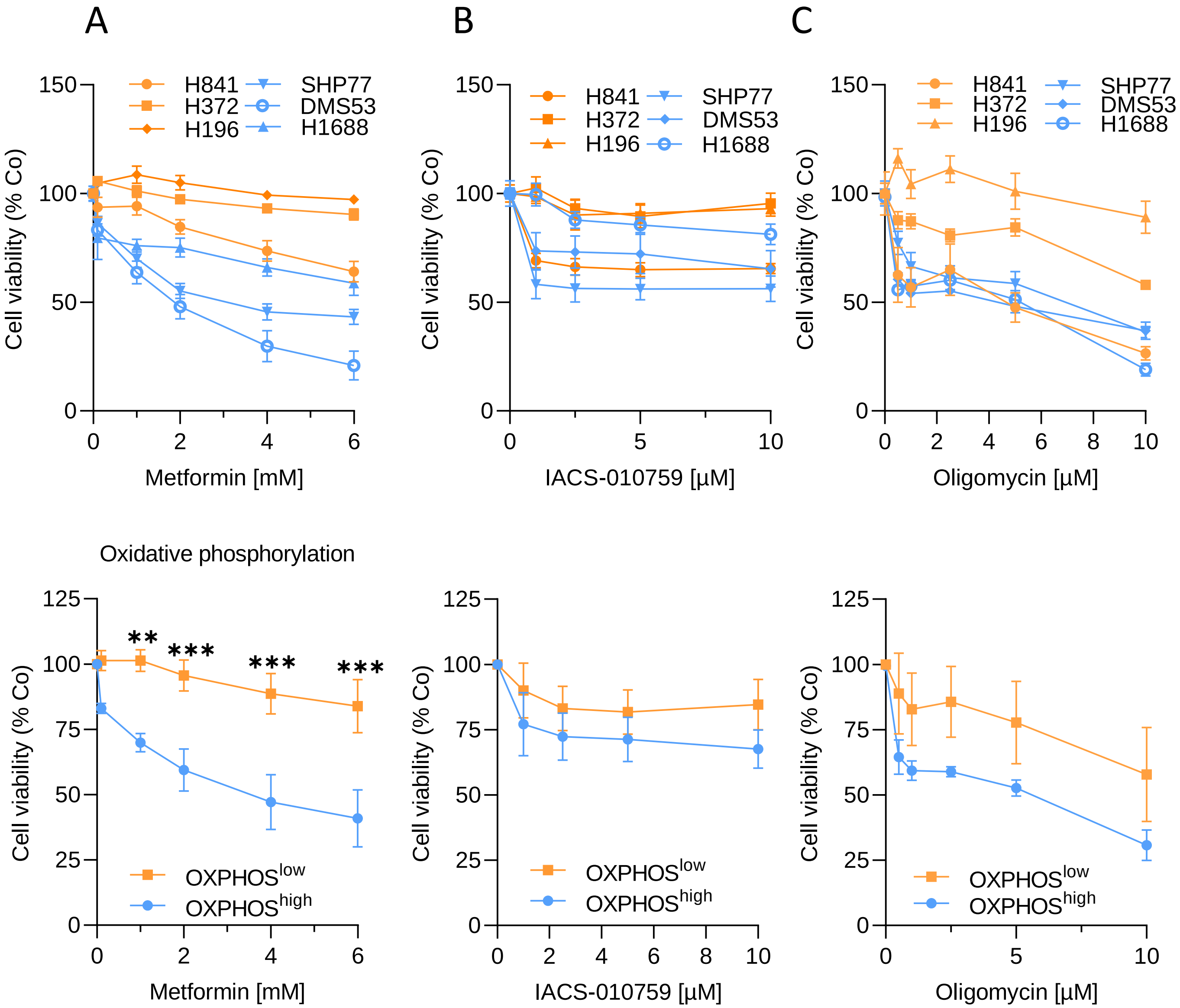

Supplementary Figure S9. Screening of metabolic inhibitors reveals differential responses between cell lines with OXPHOS^{high} or OXPHOS^{low} background. Cell viability of three OXPHOS^{high} (blue) and OXPHOS^{low} (orange) cell lines after 72 h using increasing doses of (A) metformin, (B) IACS-010759 or (C) oligomycin. Corresponding graphs at the bottom display pooled results according to group assignment (OXPHOS^{high} - blue and OXPHOS^{low} - orange). Statistical significance was determined using 2-way ANOVA and Sidak´s multiple comparisons test. ** $p < 0.01$, *** $p < 0.001$. Data are represented as mean ± SEM.

Solta et al. Supplementary Figure S9

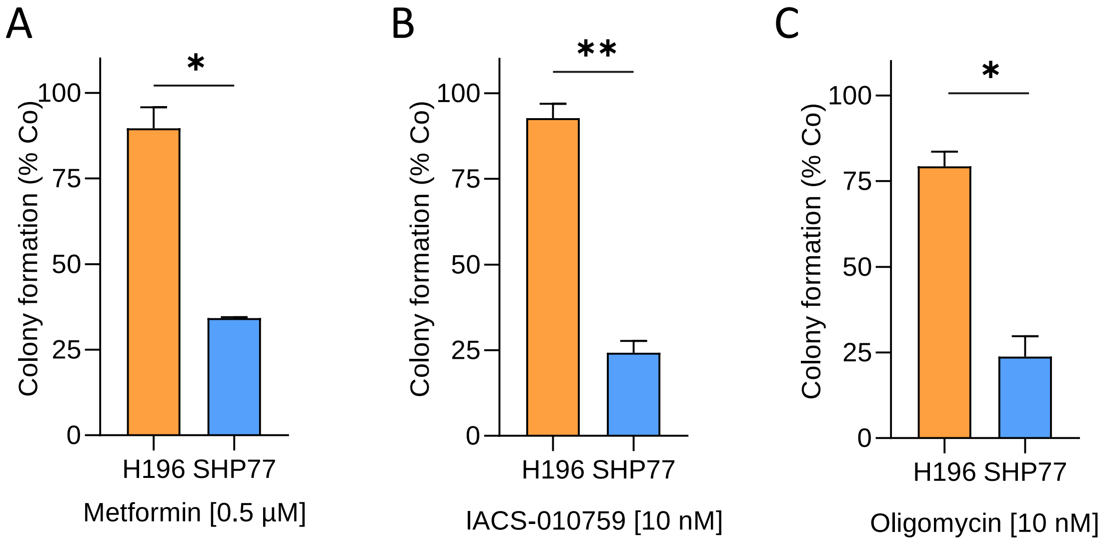

Supplementary Figure S10. SCLC-A cells are more susceptible to long-term inhibition of the electron transport chain. Longitudinal assessment of low-dose (A) metformin, (B) IACS-010759, and (C) oligomycin treatment results in less viability in SHP77 ($OXPHOS^{high}$ - blue) SCLC cells compared to H196 ($OXPHOS^{low}$ - orange). Colony formation was determined using crystal blue stainings. Statistical significance was calculated using Mann-Whitney tests. * $p < 0.05$, ** $p < 0.01$. Data are represented as mean ± SEM.

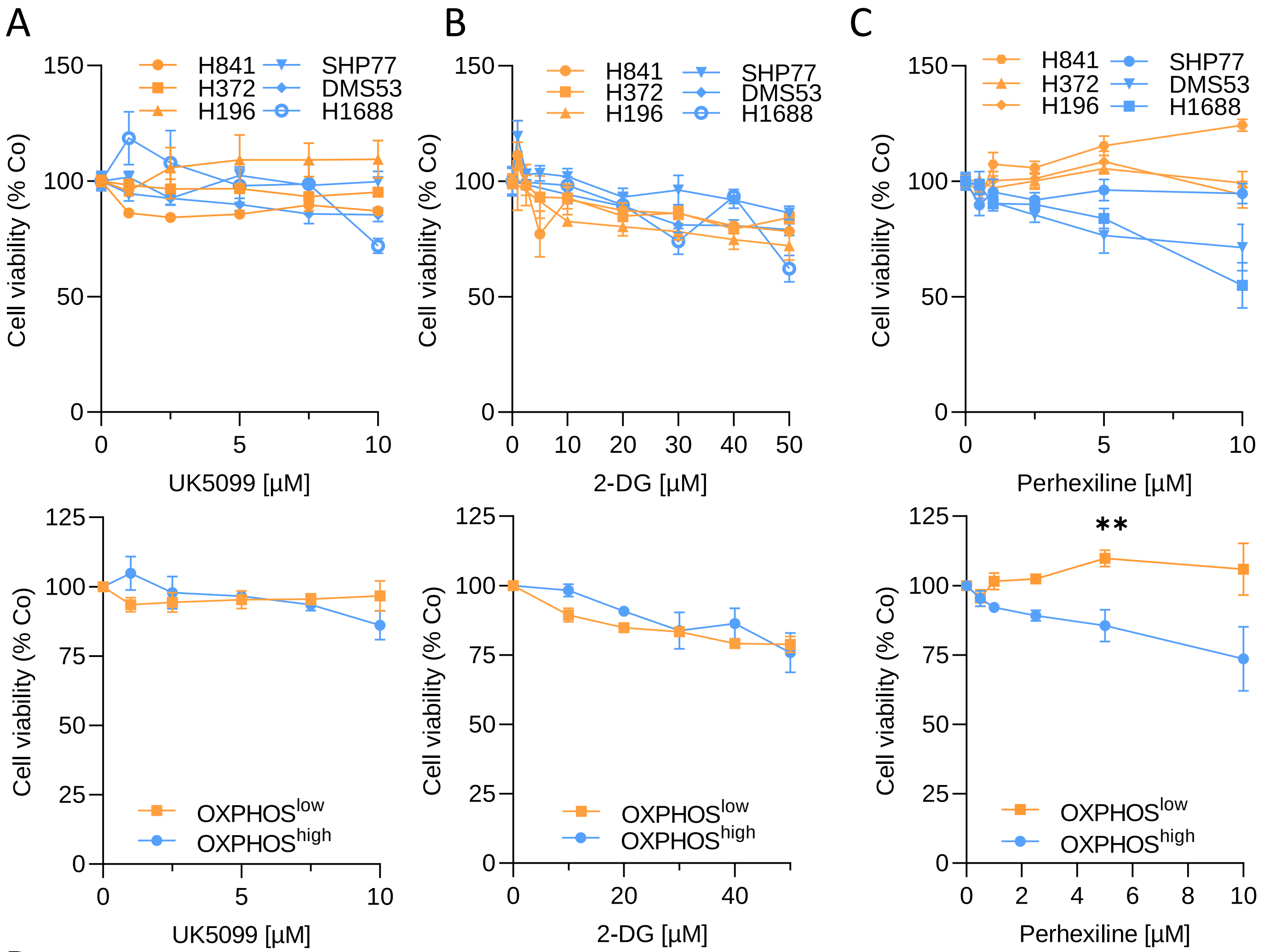

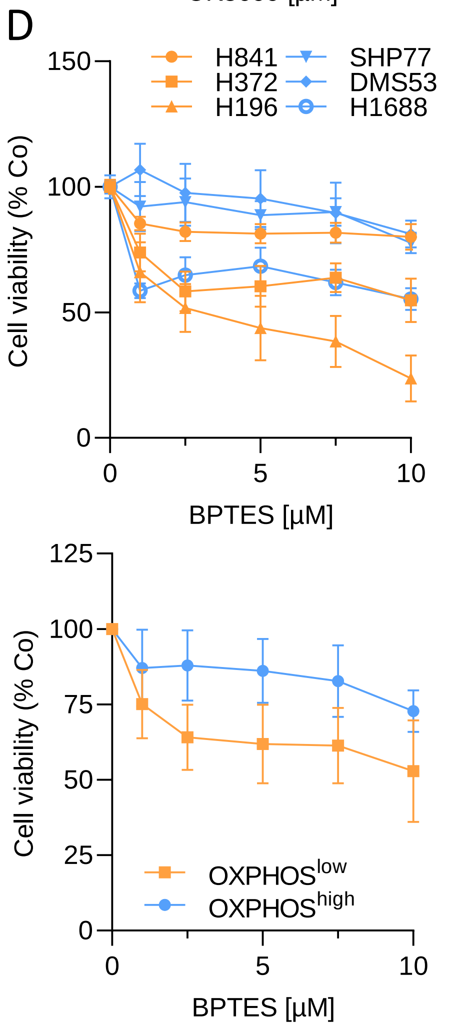

Supplementary Figure S11. Screening of metabolic inhibitors reveals differential responses between cell lines with different metabolic signatures. Cell viability of three OXPHOS^{high} (blue) and OXPHOS^{low} (orange) cell lines after 72 h using increasing doses of (A) UK-5099, (B) 2-Deoxy-D-glucose, (C) Perhexiline or (D) BPTES was determined using an MTT-based approach. Corresponding graphs display pooled results according to group assignment (OXPHOS^{high} - blue and OXPHOS^{low} - orange).Statistical significance was determined using 2-way ANOVA and Sidak´s multiple comparisons test. ** $p < 0.01$. Data are represented as mean ± SEM.

Solta et al. Supplementary Figure S11

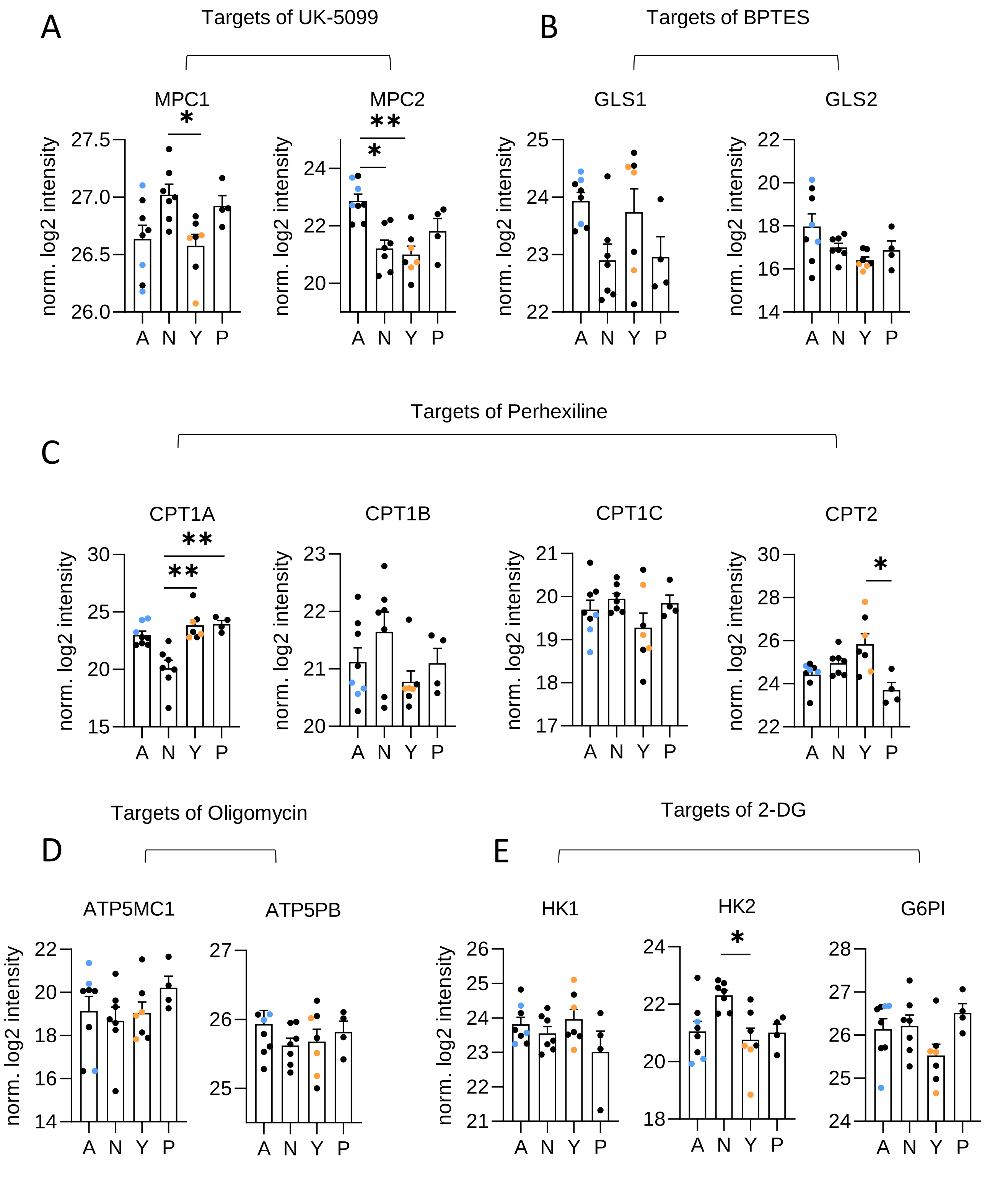

Supplementary Figure S12. Protein intensities according to proteomic data. Values are shown as normalized log2 intensity and each dot represents one SCLC cell line. Cell lines were assigned based on the molecular subtypes and cell line panels are shown in blue (SCLC-A) and orange (non-SCLC-A). ** p<0.01. (A) MPC1 and MPC2 are targets of the mitochondrial pyruvate carrier inhibitor UK-5099. (B) GLS1 and GLS2 represent targets of the glutaminolysis inhibitor BPTES. (C) CPT1A/B/C an CPT2 are inhibited by perhexiline, reducing fatty acid metabolism. (D) Oligomycin inhibits the F(0) part of the H^+-ATP-synthase (proteomics contained ATP5MC1 and ATP5PB). (E) 2-Deoxy-D-glucose is a competitive inhibitor of glucose metabolism affecting HK1, HK2 and G6PI. One-way ANOVA following Kuskall-Wallis test. * p<0.05, ** p<0.01. Data are represented as mean ± SEM.

Solta et al. Supplementary Figure S12

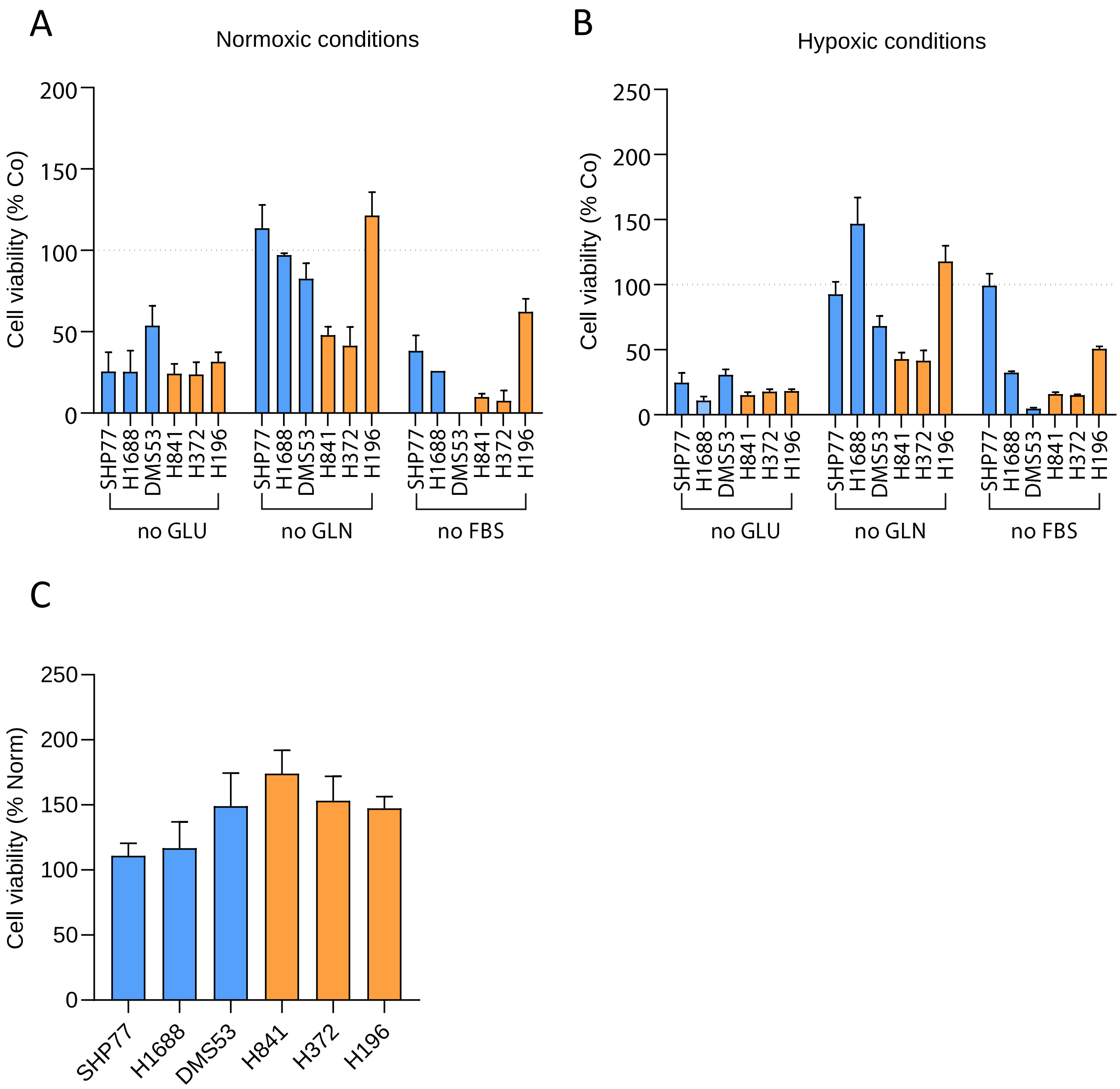

Supplementary Figure S13. Hypoxic conditions and absence of l-glutamine influenced cell viabiliy in almost all SCLC cell lines. Experiments using modified media without glucose (no GLU), l-glutamine (no GLN) or fetal bovine serum (no FBS) under (A) normoxic and (B) hypoxic conditions. (C) Percent cell viability of the cell line panels ($OXPHOS^{high}$ - blue; $OXPHOS^{low}$ - orange) under hypoxic conditions compared to corresponding normoxic controls after 72 h. Data are represented as mean ± SEM.

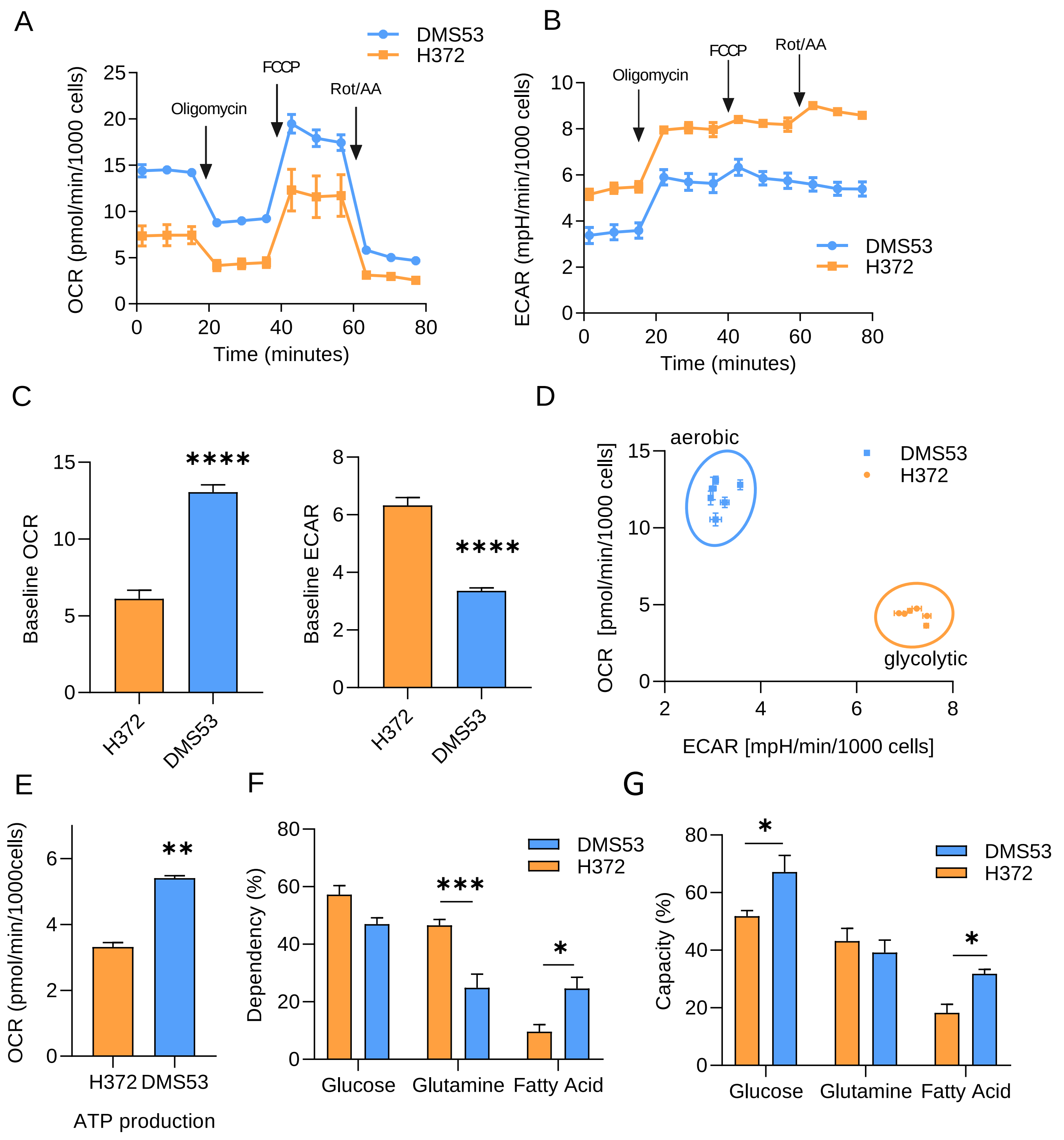

Supplementary Figure S14: Mitochondrial metabolism is superior in OXPHOS^{high} cells that are more dependent on fatty acid synthesis. Seahorse Mito Stress Test results showing (A) mitochondrial oxygen consumption rates (OCR) and (B) extracellular acidification rates (ECAR) of the OXPHOS^{high} DMS53 (blue) and the OXPHOS^{low} H372 (orange) cell lines. (C) Comparison of baseline oxygen consumption rates (OCR) and extracellular acidification rates (ECAR). Mann-Whitney test, **** $p < 0.0001$. (D) Energy map of ECAR versus OCR depicting corresponding metabolic states (aerobic: upper left; glycolytic: lower right). (E) ATP-linked respiration calculated by the decrease in OCR following ATP synthase inhibition (oligomycin) in DMS53 (blue) and H372 (orange) cells. Mann-Whitney test, ** $p < 0.01$. Results obtained from Seahorse Fuel Flex Test depicting (F) metabolic dependency and (G) capacity of DMS53 (blue) and H372 (orange) cell lines. Two-way ANOVA and Sidak´s multiple comparisons test. * $p \leq 0.05$, ** $p < 0.01$, *** $p < 0.001$. Data are represented as mean ± SEM.

Solta et al. Supplementary Figure S14

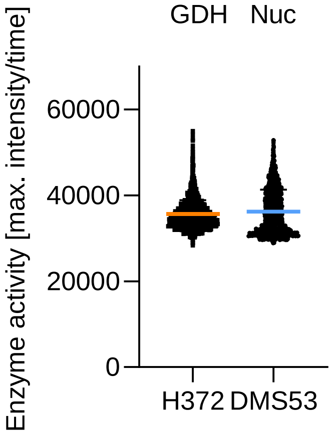

Supplementary Figure S15. Single-cell enzyme activity assays of nuclear GDH. Nuclear activities (single-nuclei) of GDH in DMS53 (OXPHOS^{high}, blue) and H372 (OXPHOS^{low} orange).

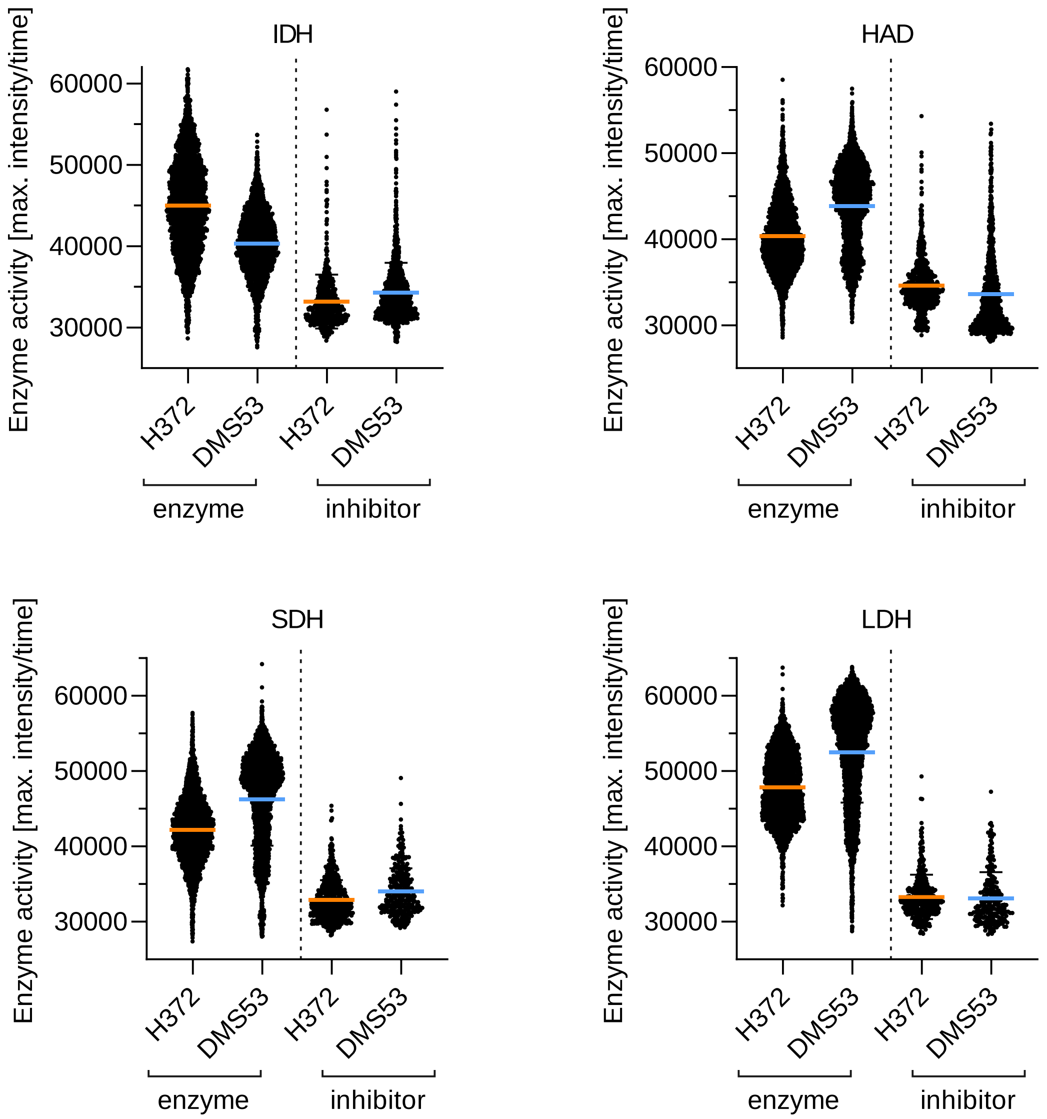

Supplementary Figure S16. Single-cell enzyme activity assays revealed differential activity of nuclear GDH and total IDH, HAD, SDH and LDH in SCLC cell lines. (A) Nuclear activities (single-nuclei) of GDH in DMS53 ($OXPHOS^{high}$, blue) and H372 ($OXPHOS^{low}$, orange). (B) Enzymatic activity of IDH, HAD, SDH and LDH of DMS53 ($OXPHOS^{high}$, blue) and H372 ($OXPHOS^{low}$, orange) at single-cell level.

Solta et al. Supplementary Figure S16

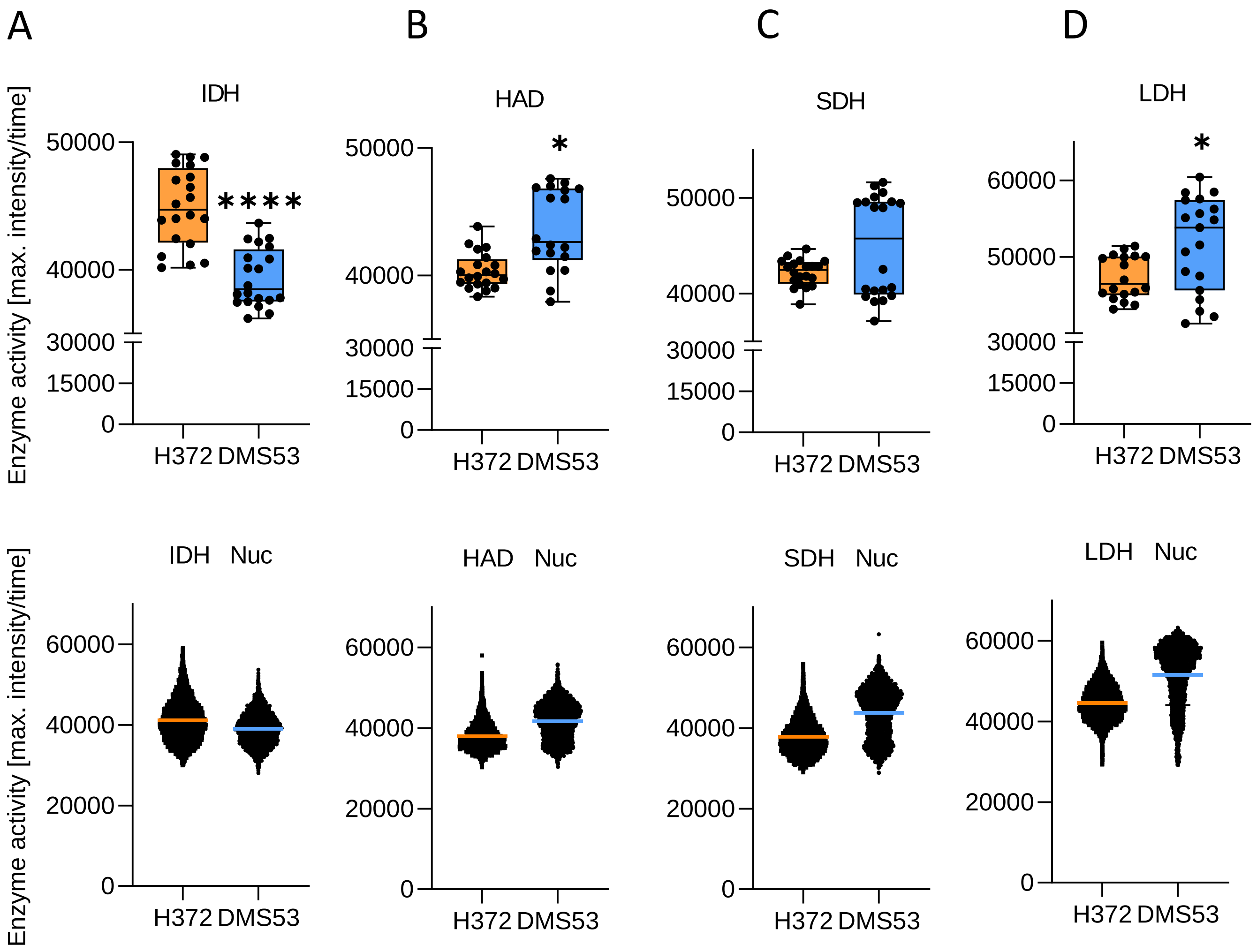

Supplementary Figure S17. Evaluation of enzymehistochemical stainings of representative SCLC cell lines. Overall enzymatic activities (mean per image) and nuclear activities (single-nuclei) of (A) IDH, (B) HAD, (C) SDH and (D) LDH. The max. enzyme activity per image acquired were analyzed for pairwise comparison (n=20 images per group, Mann-Whitney Test, * $p<0.05$, **** $p<0.0001$). Analysis of staining intensity with regard to nuclear area is depicted. Data are represented as mean ± SEM.

Solta et al. Supplementary Figure S17

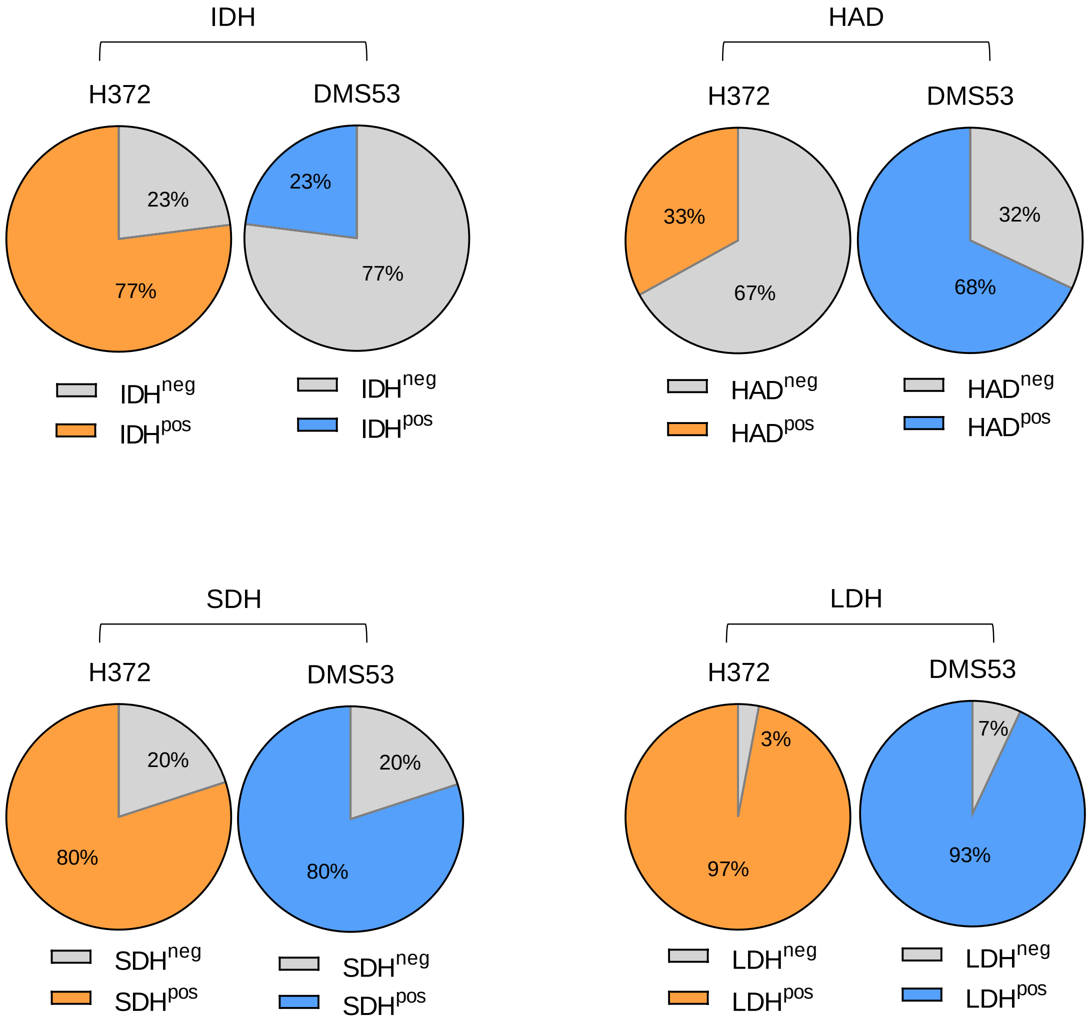

Supplementary Figure S18. Single-cell enzyme activity assays revealed differential activity of IDH, HAD, SDH and LDH in SCLC cell lines. Frequency distribution (cut-off defined by respective negative inhibitor stainings, 97.3%) in DMS53 ($OXPHOS^{high}$, blue) and H372 ($OXPHOS^{low}$, orange) cells.

Solta et al. Supplementary Figure S18

A

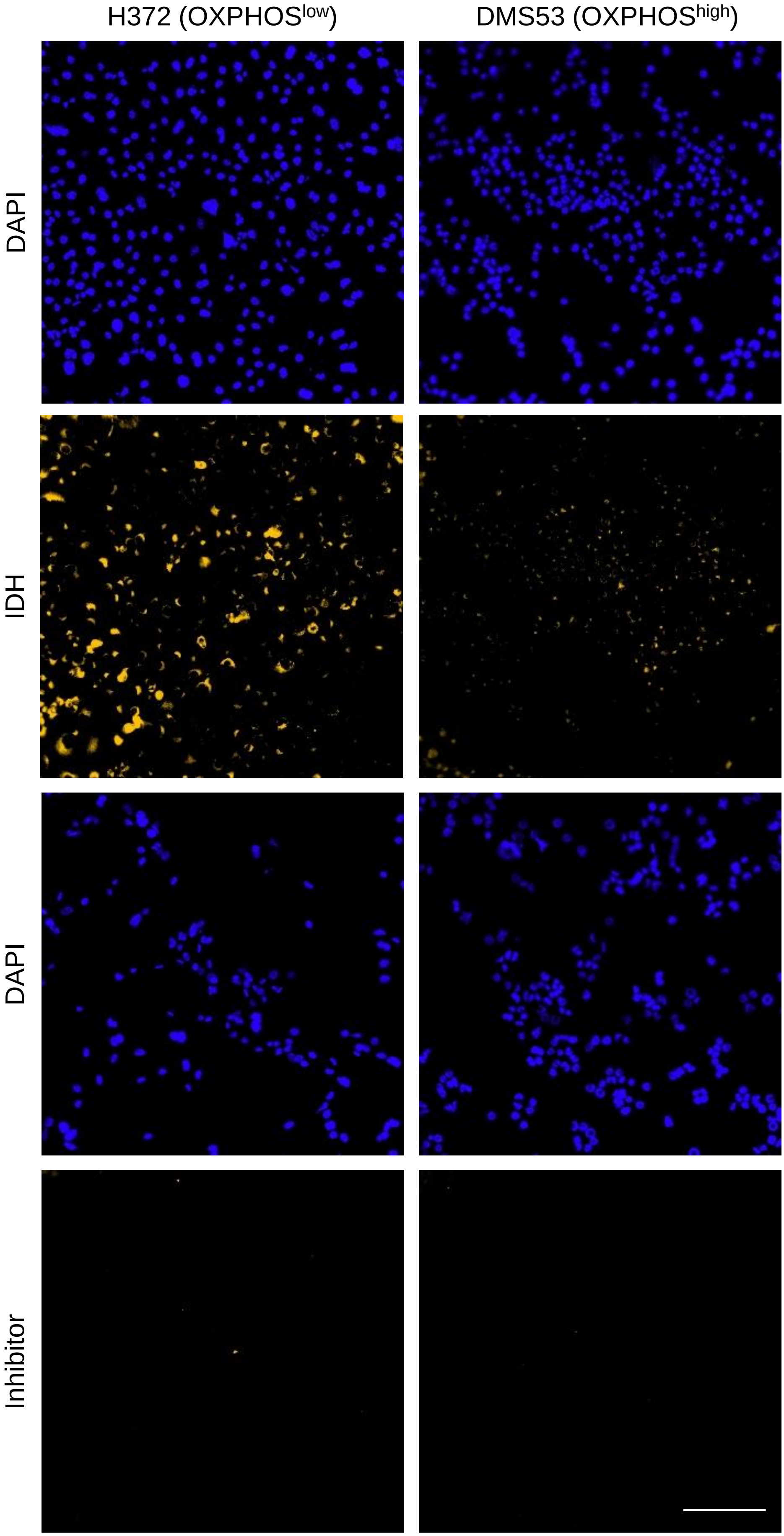

Solta et al. Supplementary Figure S19

B

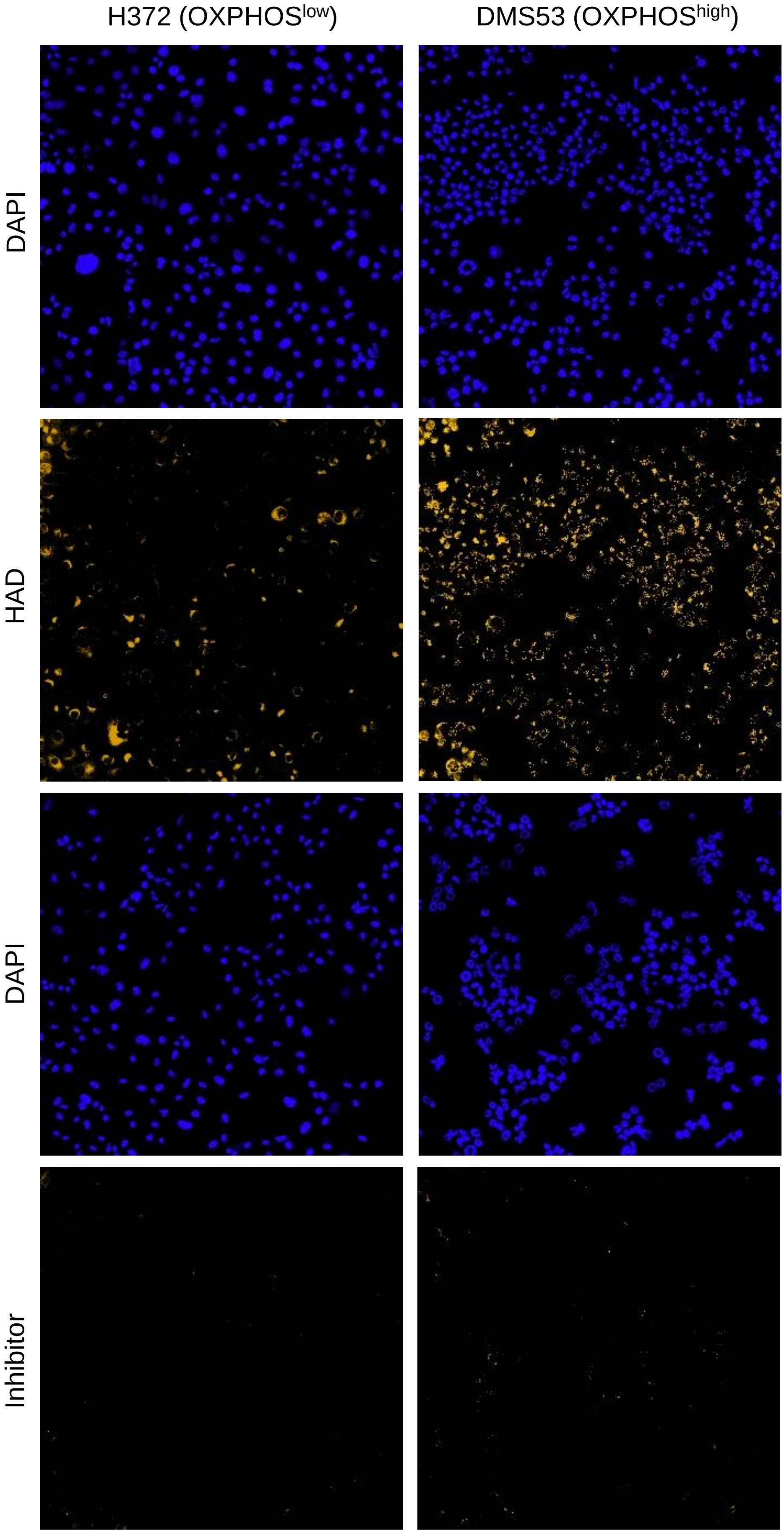

Solta et al. Supplementary Figure S19 cont.

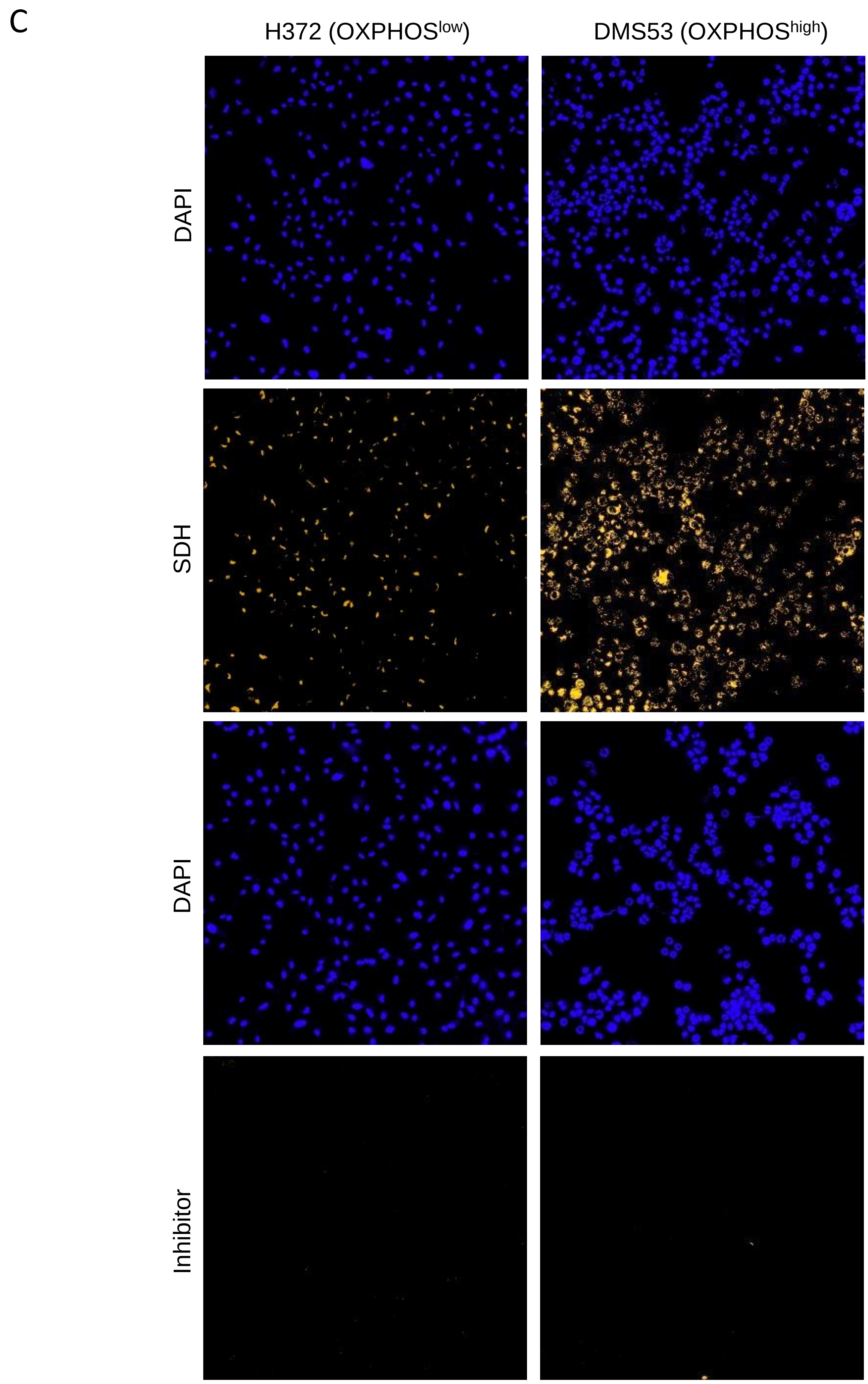

Solta et al. Supplementary Figure S19 cont.

D

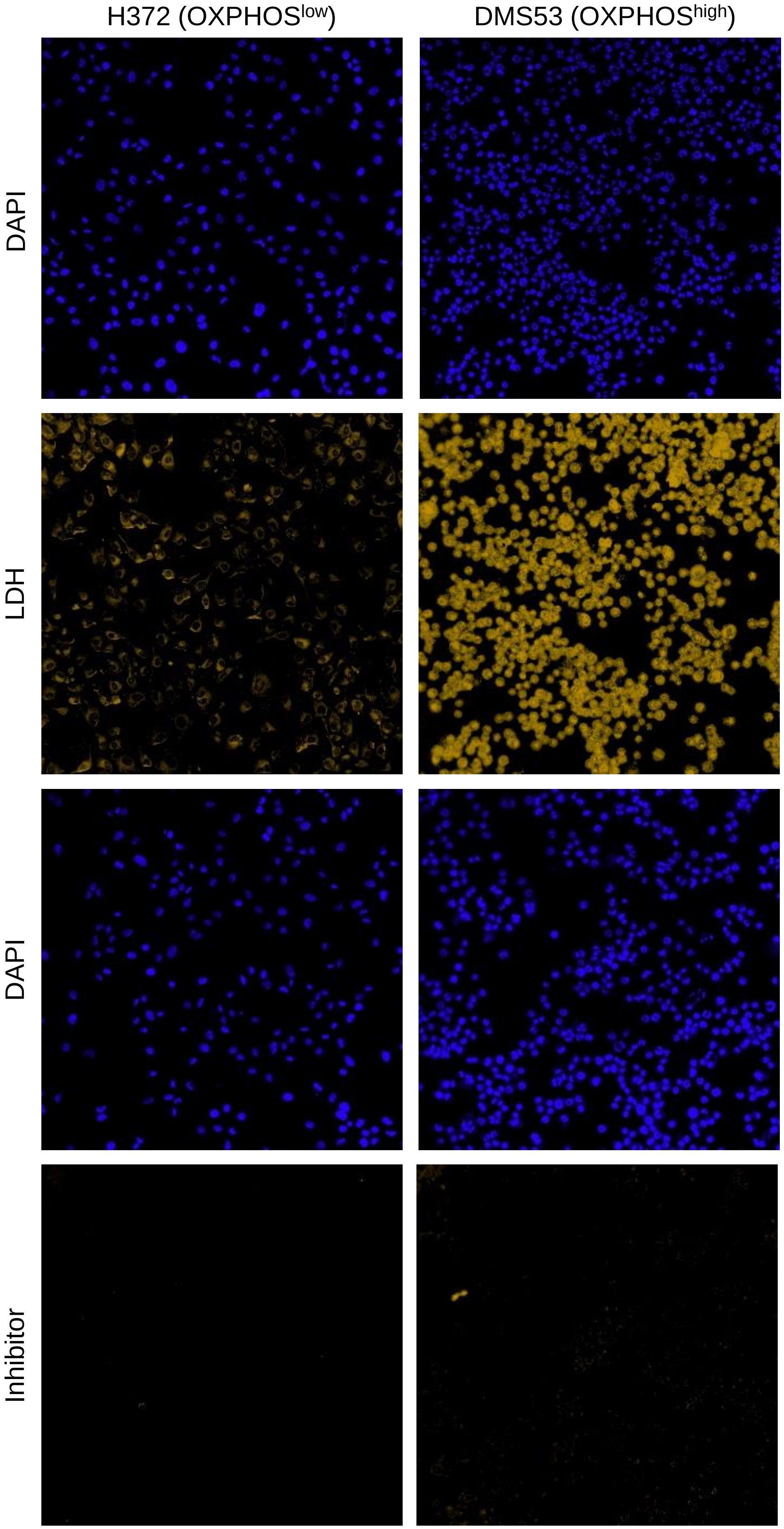

Solta et al. Supplementary Figure S19 cont.

Supplementary Figure S19. Enzymehistochemical stainings of representative SCLC cell lines. Enzymatic activities and corresponding inhibitor reactions of (A) IDH, (B) HAD, (C) SDH and (D) LDH (yellow stainings). Images were acqired with a 20x objective. Scale bar: 100 µm.

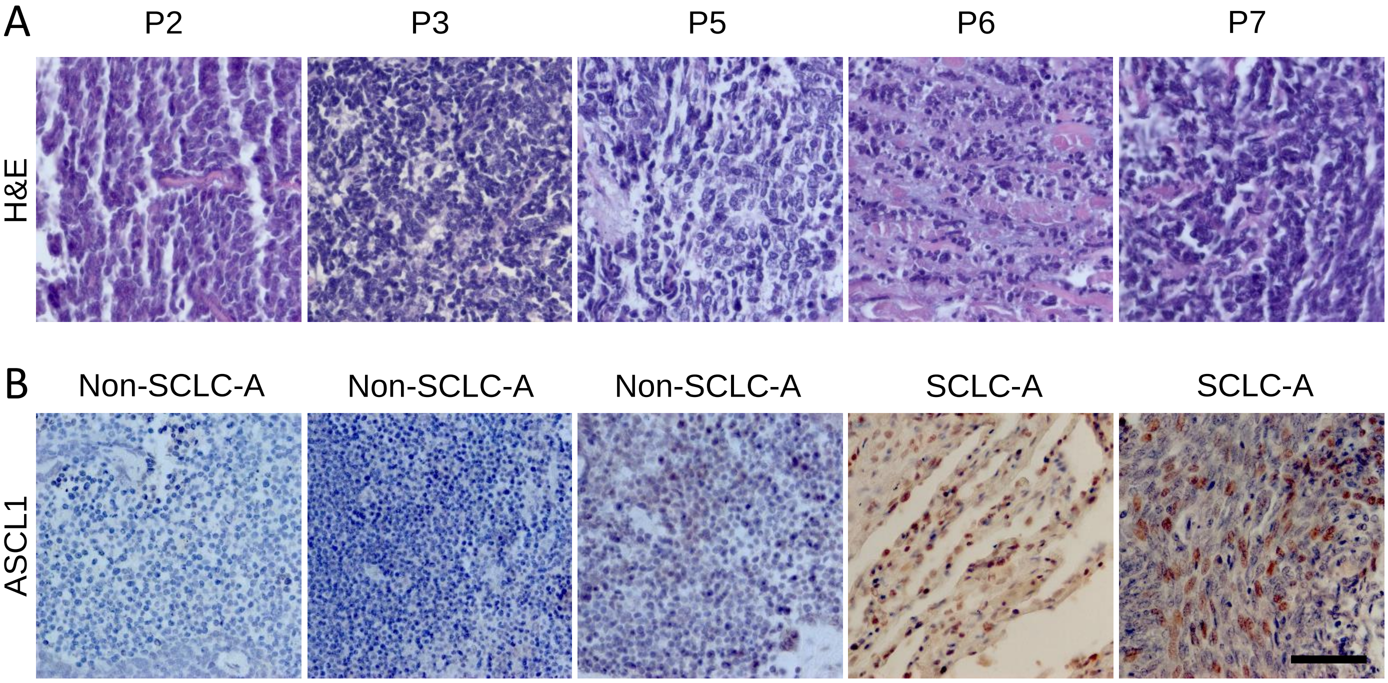

C

Sample	ASCL1	NeuroD1	POU2F3	YAP1
P7	80%	20%	0%	0%
P6	40%	35%	5%	0%
P3	0%	75%	20%	0%
P5	5%	30%	20%	10%
P2	0%	15%	10%	0%

Supplementary Figure S20. Immunohistochemical staining of primary tumors derived from rapid autopsy samples. (A) H&E staining and (B) expression patterns of the transcription factor ASCL1 are depicted. All images were acquired with a 40x objective. Scale bar: 50 µm. (C) Expression patterns of the transcription factors ASCL1, NEUROD1, POU2F3, and YAP1 of rapid research autopsy samples defined by indepenend pathologists.

Solta et al. Supplementary Figure S20

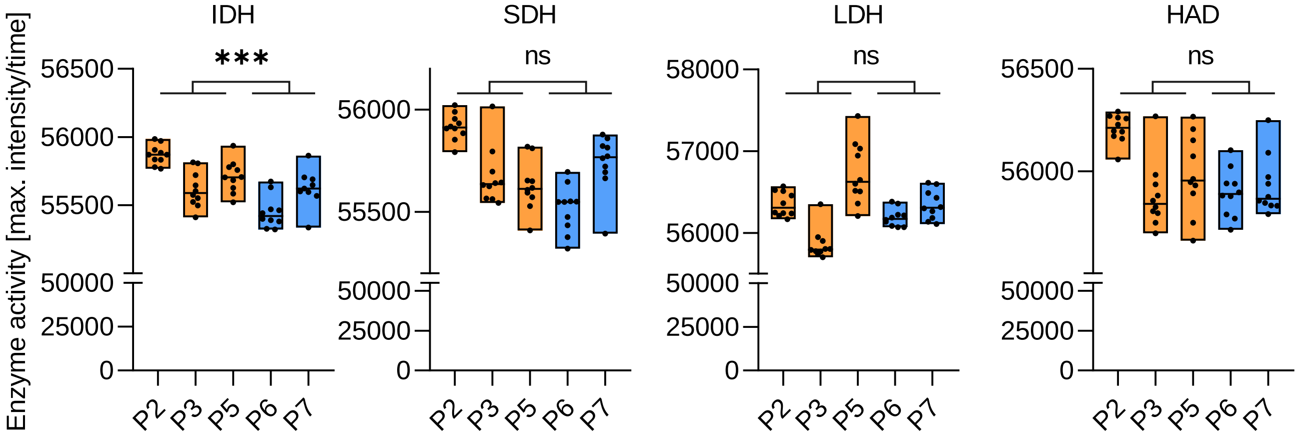

Supplementary Figure S21. Enzyme activity in primary tumors derived from rapid autopsy samples. Average positive intensity of IDH, SDH, LDH, and HAD (left to right) in snap frozen primary tumors of SCLC patients. Orange and blue color indicate non-SCLC-A and SCLC-A-dominant phenotypes, respectively.

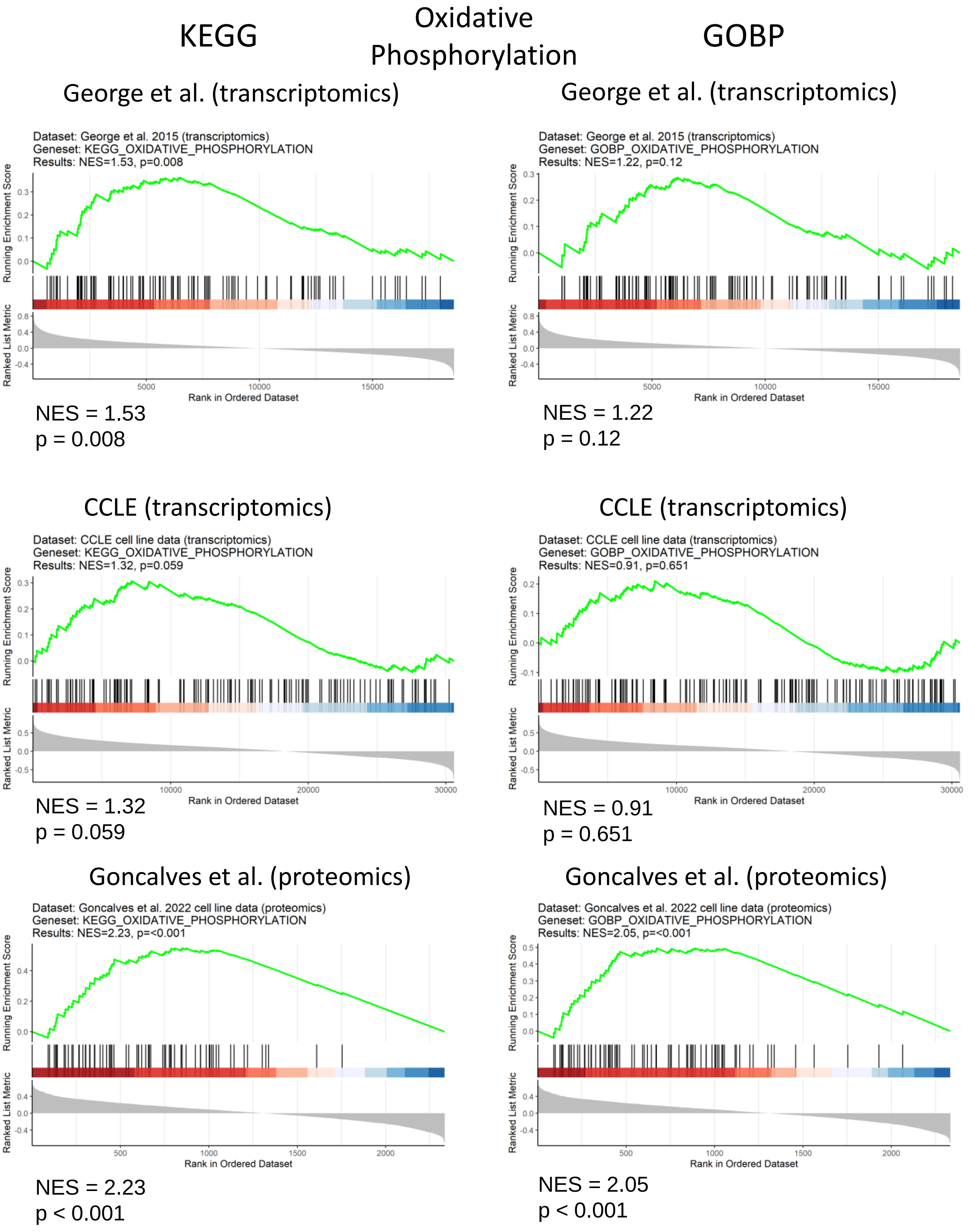

Supplementary Figure S22. Gene set enrichment analyses (GSEA) of publicly available data. Enrichment plots of pre-ranked GSEA for KEGG (left) and GOBP (right) Oxidative phosphorylation genesets in SCLC-A compared to non-SCLC-A. The y-axis indicates the normalized enrichment score (NES).
